# Comparative analysis of sequence features involved in the recognition of tandem splice sites

**DOI:** 10.1186/1471-2164-9-202

**Published:** 2008-04-30

**Authors:** Ralf Bortfeldt, Stefanie Schindler, Karol Szafranski, Stefan Schuster, Dirk Holste

**Affiliations:** 1 Department of Bioinformatics, Friedrich-Schiller University, Ernst-Abbe-Platz 2, D-07743 Jena, Germany; 2 Fritz-Lipmann Institute for Aging Research, Beutenbergstraße 11, D-07745 Jena, Germany; 3 Research Institute of Molecular Pathology, Dr. Bohr-Gasse 7, A-1030, Vienna, Austria; 4 Institute of Molecular Biotechnology of the Austrian Academy of Sciences, Dr. Bohr-Gasse 3-5, A-1030, Vienna, Austria

## Abstract

**Background:**

The splicing of pre-mRNAs is conspicuously often variable and produces multiple alternatively spliced (AS) isoforms that encode different messages from one gene locus. Computational studies uncovered a class of highly similar isoforms, which were related to tandem 5'-splice sites (5'ss) and 3'-splice sites (3'ss), yet with very sparse anecdotal evidence in experimental studies. To compare the types and levels of alternative tandem splice site exons occurring in different human organ systems and cell types, and to study known sequence features involved in the recognition and distinction of neighboring splice sites, we performed large-scale, stringent alignments of cDNA sequences and ESTs to the human and mouse genomes, followed by experimental validation.

**Results:**

We analyzed alternative 5'ss exons (A5Es) and alternative 3'ss exons (A3Es), derived from transcript sequences that were aligned to assembled genome sequences to infer patterns of AS occurring in several thousands of genes. Comparing the levels of overlapping (tandem) and non-overlapping (competitive) A5Es and A3Es, a clear preference of isoforms was seen for tandem acceptors and donors, with four nucleotides and three to six nucleotides long exon extensions, respectively. A subset of inferred A5E tandem exons was selected and experimentally validated. With the focus on A5Es, we investigated their transcript coverage, sequence conservation and base-paring to U1 snRNA, proximal and distal splice site classification, candidate motifs for *cis*-regulatory activity, and compared A5Es with A3Es, constitutive and pseudo-exons, in *H. sapiens *and *M. musculus*. The results reveal a small but authentic enriched set of tandem splice site preference, with specific distances between proximal and distal 5'ss (3'ss), which showed a marked dichotomy between the levels of in- and out-of-frame splicing for A5Es and A3Es, respectively, identified a number of candidate NMD targets, and allowed a rough estimation of a number of undetected tandem donors based on splice site information.

**Conclusion:**

This comparative study distinguishes tandem 5'ss and 3'ss, with three to six nucleotides long extensions, as having unusually high proportions of AS, experimentally validates tandem donors in a panel of different human tissues, highlights the dichotomy in the types of AS occurring at tandem splice sites, and elucidates that human alternative exons spliced at overlapping 5'ss posses features of typical splice variants that could well be beneficial for the cell.

## Background

As the central intermediate between transcription and translation of eukaryotic genes, the splicing of precursors to messenger RNAs (pre-mRNAs) in the nucleus is frequently variable and produces multiple alternatively spliced (AS) mRNA isoforms. The recognition of authentic pre-mRNA splice sites out of many possible pseudo-sites, the precise excision of introns, and the ligation of exons to produce a correct message are catalyzed by a large ribonucleoprotein (RNP) complex known as the spliceosome, which is composed of several small RNPs and perhaps over two-hundred proteins [1]. Splice sites mark the boundaries between exon and intron: a 5'-splice site (5'ss or donor) at the terminus of the exon/beginning of the intron and a 3'ss (acceptor) at the terminus of the intron/beginning of the exon. In addition, introns contain a branch point signal, typically 15 to 45 nucleotides upstream of the 3'ss. During later stages of spliceosome assembly, there are mediated interactions between the 5'ss and 3'ss, as well as splicing factors that recognize them, and a basic distinction is made between the pairing of splice sites across the exon ('exon-definition') or the intron ('intron-definition') [2]. In humans, with compact exons (average length of about 120 nucleotides) and comparatively much larger introns, exon-definition is thought to be the prevalent mode of RNA splicing. When a pair of closely spaced 3'ss-5'ss signals is recognized, the exon is roughly defined by interactions between U2 snRNP:3'ss, U1 snRNP:5'ss as well as additional splicing factors, including U2AF_65_:branch site and U2AF_35_:poly-(Y) site interactions.

AS events are categorized according to their splice site choice and one can distinguish four canonical types: exon-skipping (SE), in which mRNA isoforms differ by the inclusion/exclusion of an exon; alternative 5'ss exon (A5E) or alternative 3'ss exon (A3E), in which isoforms differ in the usage of a 5'ss or 3'ss, respectively; and retention-type intron (RI), in which isoforms differ by the presence/absence of an unspliced intron [3]. These types are not necessarily mutually exclusive and more complex types of AS events can be constructed from such canonical types. Alternative splicing produces similar, yet different messages from one gene locus, thus enabling the diversification of protein sequences and function [4]. In addition, AS holds the possibility to control gene expression at the post-transcriptional level via the non-sense mediated mRNA decay (NMD) pathway. To prevent aberrantly or deliberately incorrectly spliced transcripts that prematurely terminate translation, NMD ensures that only correctly spliced mRNAs that contain the full (or nearly so) message are subsequently utilized for protein synthesis. Therefore, NMD scans newly synthesized mRNA for the presence of one or more premature-termination codons (PTCs), and, if detected, can selectively degrade defective mRNAs [5].

Fostered by the abundant accumulation of complementary DNA (cDNA) sequences and expressed sequence tags (ESTs), genome-wide computational studies of AS have investigated its scope in metazoans and estimated that a fraction of up to two-thirds of human genes are predicted to encode or regulate protein synthesis via such pathways [6-9]. The outcome of these approaches have shown SEs as the most frequent AS event in mRNA isoforms in human and other mammalian organ systems and cell types, followed by A3Es and A5Es, in turn followed by RIs [10]. Interestingly, the sequence information of SEs and their flanking regions, and the phylogenetic conservation of such information, is sufficient to discriminate constitutive exons from SEs and can be used in computational models to start predicting AS events that have not yet been uncovered by cDNA and EST analyses [11,12].

Compared with the skipping of about one hundred exon nucleotides or the retention of several hundred intron nucleotides, A3Es and A5Es are thought to create more subtle changes, by affecting the choice of the 3'ss or 5'ss, respectively. Here, splice site usage gives rise to two types of exon segments – the 'core' common to both splice forms and the 'extension' that is present in only the longer isoform. Both types of AS events have been shown to play decisive roles during development (e.g., sex determination and differentiation in *Drosophila melanogaster *[13] or developmental stage-related changes in the human *CFTR *gene [14]), but also in human disease (e.g. 5'ss mutations in the *tau *gene [15]). A3Es and A5Es are thought to be regulated by splicing-regulatory elements in exons and nearby exon-flanking regions, as well as *trans*-acting antagonistic splicing factors, which bind them and affect the choice of splice sites in a concentration dependent manner [16,17]. Interestingly, computational studies showed that for both A3Es and A5Es the distribution of extensions, *f*(*E*), is markedly skewed toward short-range splice forms [18]. In particular, alternative splice sites that are separated by the three-nucleotide long motif NAG/NAG/(where '/' marks an inferred splice site) make up a predominant proportion of A3E events in a mammals, extending to invertebrates and plants [19,20]. Yet additional support from experimental studies is still very sparse, and the similarities and dissimilarities of overlapping against non-overlapping ("competitive") as well as constitutive splice sites remain to be delineated.

Here, we describe an effort to compare and contrast A5E, A3E, and constitutive splice sites of human exons derived from transcript sequences, of different human organ systems and cell types, which were aligned to the assembled human genome sequence. To study known sequence features involved in the recognition and distinction of splice sites, we performed large-scale but stringent alignments of cDNAs and ESTs to the human and mouse genome. Subsequently, we experimentally validated a subset of computationally inferred patterns of overlapping AS patterns, by RT-PCR and direct sequencing, analyzed implicated sequence and transcript features, and compared A5Es with constitutive and pseudo-exons, as well as A3Es, in *H. sapiens *and *M. musculus*. We found differences for sequence conservation and base-pairing to U1 snRNA, proximal/distal splice site utilization, occurrence of candidate motifs, and transcript coverage in subsets of overlapping 5'ss.

Our results distinguish a small but authentic enriched set of A5Es (A3Es), with specific distances between proximal and distal 5'ss (3'ss), which show a marked dichotomy between the levels of in- and out-of-frame tandem splice site usage, identify a number of candidate NMD targets, and allow the rough estimation of a number of unobserved tandem AS events based on splice site information. The implications for the processing of human alternative transcripts are discussed.

## Results

### Biased extensions of alternative 5'ss and 3'ss exons

Exon-skipping is the most prevalent AS type produced by the human spliceosome, as well as by all other mammals investigated to date, when averaged across different organ systems and cell types that can exhibit tissue-enriched splice forms [21,22]. Internal alternative exons that involve exclusively either the 3'ss (A3Es) or the 5'ss (A5Es) are also abundantly produced, while the simultaneous alteration of 3'ss and 5'ss (producing exons that overlap but match neither splice site) are markedly less frequent. For A5Es the most distal splice site defines the exon core, while proximal sites (if more than one alternative choice is possible) are exon extensions only included in selected mRNAs.

Out of a collection of ~37,400 transcript-inferred human alternative exons maintained in the HOLLYWOOD database [23], AS events of about 10,300 A5Es and 9,200 A3Es were filtered for exon splice variants of solely one proximal/one distal 5'ss, while being constitutively spliced at the opposite site, and resulted to 5,275 A5Es and 4,497 A3Es; either exon set had no other inferred AS type, respectively. Stringent alignment criteria were imposed on all transcripts: 1) ESTs were required to overlap at least one co-aligned cDNA; 2) the first and last aligned segments of ESTs were required to be at least 30 nucleotides in length with 90% sequence identity; 3) the entire EST sequence alignment was required to extend over at least 90% of the length of the EST with at least 90% sequence identity; and 4) realignments of ESTs with two other algorithms were required to agree in three out of all three independent alignments (see below, as well as Methods). The resulting dataset of identical computational inferences of three methods contained 1,868 (~18%) A5Es and 3,301 (~36%) A3Es.

**Figure 1:**
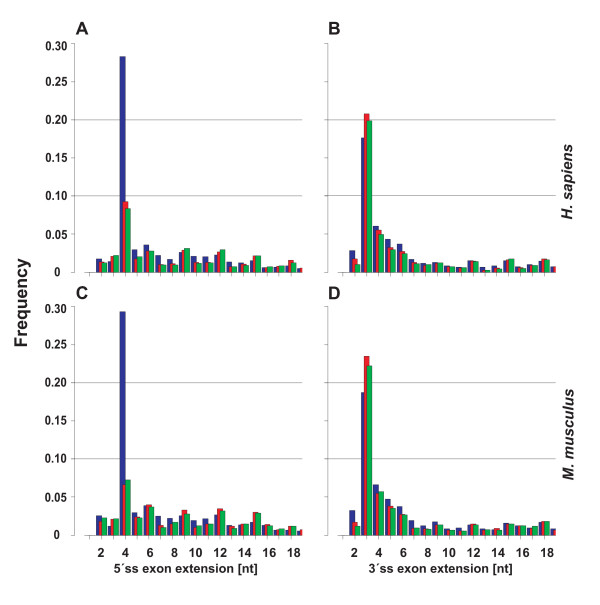
**Occurrence of extensions (*E *= 1,2,...,18 nucleotides) for A5Es (parts A, C) and A3Es (B, D), with human and mouse exons in the top and bottom panels, respectively.** Extensions were inferred from three different alignment algorithms (colored as blue, SIM4; red, BLAT; and green, EXALIN) of cDNAs/ESTs to genomic DNA. The distribution *f*(*E*) for A5Es was markedly biased for extensions (*E*) with overlapping splice sites, with a peak at *E *= 4 nucleotides. Exon extensions exhibited relatively smaller but persistent periodic peaks at *E *= 6, 9, 12, 15, and 18 nucleotides. *f*(*E*) for A3Es also displayed a bias for overlapping splice sites, with a peak at *E *= 3 nucleotides and smaller peaks at 4–6 nucleotides. The program SIM4 predicted significantly more extensions at *E *= 4 nucleotides as compared to BLAT and EXALIN predictions of the same initial set of cDNAs/ESTs, which was indicative of spurious alignments. A comparative analysis of alternative exons in *M. musculus *corroborated the above patterns.

We subdivided alternative exons into their core and extension, where the latter is the sequence between the distal and proximal splice sites. The extension (*E*) included lengths up to about 250 nucleotides, with quickly decreasing transcript coverage/utilization as *E *increases. Larger extensions existed, albeit with barely more than a few transcripts (data not shown). For the sake of simplicity, we defined the boundary between A5E (A3E) overlapping and non-overlapping splices at *E *> 6 (*E *> 18) nucleotides and displayed the distribution *f*(*E*) for *E *= 1,2,...,18 nucleotides in a window across the boundary region. Noticeably, the obtained distribution *f*(*E*) for both A5Es and A3Es was highly biased for extensions with overlapping splice sites. Figure 1 shows (in the upper-left panel) that for extensions at the 5'ss the bias is caused predominantly by a peak at *E *= 4 nucleotides. It further shows for A5Es that short extensions exhibit a small but persistent pattern periodically occurring at *E *= 6, 9, 12, 15, and 18 nucleotides, all multiples of three, and thus preserving the reading-frame. These patterns of AS for short extensions were in accord, both qualitatively and in good approximation quantitatively, in an independent, comparative analysis for the mouse *Mus musculus *(Figure 1, lower-left panel). Overall, the median sizes of inferred alternative exons showed that SEs and A5Es tend to be shorter than CEs and A3Es, while overlapping and skewed to larger sizes [see Additional File 1, Figure S1].

Unexpectedly, Figure 1 was indicative that different splice-alignment algorithms gave rise to quite different outcomes, particularly when faced with alignments involving short extensions. Among several standard algorithms, SIM4 displayed a strong tendency toward *E *= 4 nucleotides. We took a conservative approach to substantiate the identified A5E events, by realigning all corresponding transcripts to the same genomic sequence with two other algorithms, EXALIN and BLAT (the latter lacks an explicit splice site model). The results showed that for *E *= 4 the proportion of A5E events derived from SIM4 (~28%) was markedly higher than alignments derived from EXALIN or BLAT – yet the bias for extensions was consistently shown at *E *= 4 nucleotides, though with a lower proportion of ~9% [see Additional File 1, Table S1]. Manual inspection of selected SIM4 alignments showed apparent sequence inconsistencies, when compared to the secondary alignments [see Additional File 1]. In all, 1,868 of 5,275 A5Es were taken for further analysis, where ~9% (171/1,868) accounted for *E *= 4 nucleotides extensions.

In order to compare these findings with A3E events, we obtained the distribution of short extensions and identified a similar, albeit distinctively different pattern (upper-right panel). Figure 1 shows that *f*(*E*) exhibits a clear peak at *E *= 3 nucleotides, with successively smaller peaks at *E *= 4, 5, and 6 nucleotides. Again, these AS patterns were corroborated in a comparative analysis for *M. musculus *(Figure 1, lower-right panel). The extension preference of alternative 5'ss and 3'ss exons is in accord with previous studies, where in particular *E *= 3 nucleotides for A3Es had been examined and found to obey the pattern NAG/NAG/[20,24,25].

### Tandem donors and acceptors

Patterns of A5Es and A3E extensions with overlapping splice sites are interesting in their own context, because they are 1) occurring most abundantly; 2) possibly differently regulated than non-overlapping, i.e. competitive, splice sites of alternative 5'ss and 3'ss exons [26,27]; and 3) predictive of different downstream effects of AS, resulting into preferred different modes of alternative splicing at the 5'ss (out-of-frame splicing) and the 3'ss (in-frame splicing). For overlapping 5'ss and 3'ss are mainly characterized by extensions of four and three nucleotides, respectively, hereafter we denote by "A5EΔ4" tandem donors with *E *= 4 and similarly by "A3EΔ3" tandem acceptors with *E *= 3 nucleotides. We study for tandem donors known sequence features involved in the recognition of the 5'ss, and compare them to the 3'ss of alternative and constitutive exons, including exons with pseudo donors.

**Figure 2:**
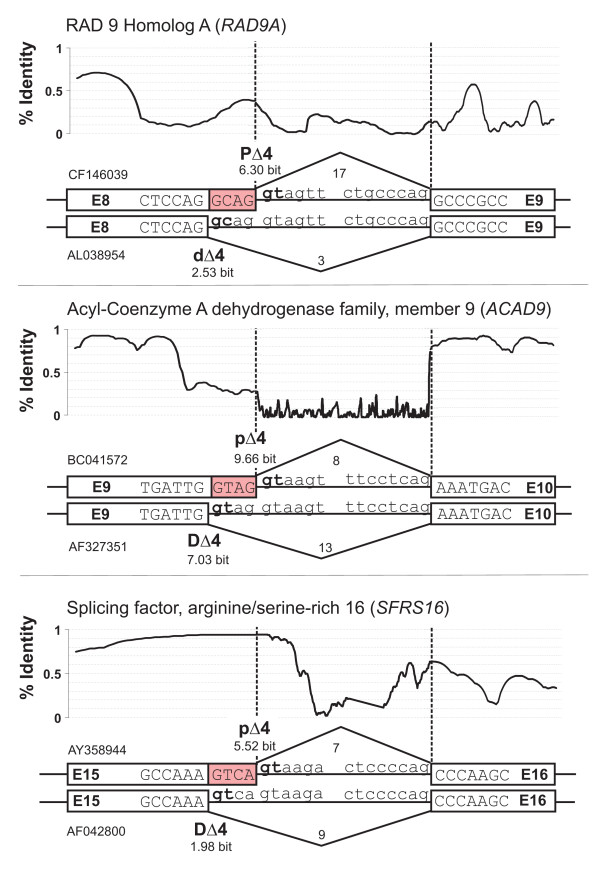
**Illustrative examples of inferred tandem donors.** White boxes denote exon and lines intron nucleotides; exon numbers (E#) corresponded to 5'-to-3' enumerated REFSEQ-annotations, the splice site score as measured by MAXENTSCAN, and the transcript coverage of the proximal and distal donor site corresponded to the number of aligned sequences. In A), E8 of the *RAD9A *gene shows a tandem donor with extension/**GCAG**/; in B) E9 of the *ACAD9 *gene shows a tandem donor with extension/**GTAG**/; in C), E15 of the *SFRS16 *gene shows a tandem donor with extension/**GTCA**/. Tandem donors in A) and C) were preferentially included in different transcripts. The conservation plot (PHASTCON scores, not in scale with the stated exon and intron nucleotides) covers A5EΔ4 splicing exons, as well as adjacent introns and downstream exons, and shows alternating patterns of high/low levels across all three examples.

Generally, the basic recognition and binding to 5'ss incorporates intronic (involving positions from 1 to 6) and exonic nucleotides (positions from -3 to -1). The consensus motif for 5'ss of mammalian genes is known as CAG/GTRAGT (at positions P_-3_P_-2_P_-1_/P_1_P_2_-P_6_), where the purine (R) is either an adenine (A) or a guanine (G) base. This nine nucleotide-long motif is highly degenerated and, in fact, in the present data set of human exons only proportions of ~0.9% (966/113,386) and ~1.3% (1,431/113,386) of inferred constitutive exons exhibited exact matches to the motifs CAG/GTAAGT or CAG/GTGAGT, respectively. Figure 2 illustrates splice sites and utilization of tandem donors for three selected human genes [see Additional file 2 for a complete list of inferred tandem donors]:

A. The gene *RAD9A *(Ensembl gene-identifier ENSG00000172613) is a homolog conserved from yeast to human, which encodes a cell cycle-check point control protein that is required for cell-cycle arrest and DNA damage repair. The primary transcript sequence of *RAD9A *exhibited two alternative, overlapping 5'ss at exon E8, identified as CAG/**GCAG**/GT at the distal 5'ss and CAG/GTAGTT at the proximal 5'ss that extends E8 (non-consensus nucleotides are underlined; exon extension bolded). The distal and proximal 5'ss gave rise to three and 17 mRNAs, respectively, which aligned to the primary transcript structure of *RAD9A*. In addition to the tandem donor pattern, Figure 2 shows the splice site strength, quantified by the MAXENT score (see Methods), and the conservation profile across exons and intron, quantified by the PHASTCON score [28] computed across several genomes (from *P. troglodytes *to *T. rubripes*). Local regions of high levels of sequence conservation for exons compared with the intron are apparent.

B. A tandem donor was detected for E9 (TTG/**GTAG**/GT and TAG/GTAAGT) of the *ACAD9 *gene (ENSG00000177646), which encodes a member of the Acyl-CoA dehydrogenase gene family and plays a role in lipid catabolism. The distal and proximal 5'ss gave rise to 13 and eight mRNAs, respectively. Figure 2 shows for E9 consistently elevated levels of sequence conservation.

C. The arginine/serine-rich splicing factor 16 (ENSG00000104859) showed a tandem donor at E15 (AAA/**GTCA**/GT and TCA/GTAAGA). Distal and proximal 5'ss choice gave rise to nine and six mRNAs of *SFRS16*, respectively. Figure 2 shows that the level of sequence conservation of E15 steadily rises toward the 3'-terminus and extends well across the exon-intron junction to I16, before it rapidly decays, which was indicative of conservation due to splicing-regulatory function [29].

### Experimental validation of tandem donors

Having obtained sufficient evidence from stringent transcript alignments, we pursued to validate the functional utilization of tandem splice sites from independent lines of evidence. To this end, we first searched publicly available literature (see Availability and requirements section for Pubmed URL) for AS events involving short 5'ss extensions. Yet we found only a very limited number of reported cases of splice variants with short extensions that could be traced back to tandem acceptors. The human *Clasp *gene (known synonyms are *SFRS16*, or *SWAP2 *for the *D. melanogaster *homolog), for instance, encodes the Clk4-associating arginine/serine-rich (SR)-related protein that binds to the family of CDC2-like kinases [30,31]. The 5'ss of E15 of the *Clasp*/*SFSR16 *is an alternative tandem donor, which gives rise to the splice forms *ClaspS *(with the extension **GTCA**) and *ClaspL *(without). Both isoforms differ by 246 nucleotides, where *ClaspS *carries a PTC due to out-of-frame splicing and thereby omits a third RS-domain encoded by *Clasp*/*SFSR16*. Both isoforms were tissue-enriched in the mice brain and testis, and displayed different intra-nuclear locations, possibly controlled by the third RS-domain [30]. Another AS event involving tandem splice sites has been detected in the human growth hormone (GH) gene cluster, whose expression is developmentally controlled. The gene *GH-V *differentially expressed three isoforms in the placenta and testis, one of which is due to a tandem donor splice site (/**GTGG**/GT) of exon E4; the tandem site was not sequence-conserved in the remaining four family members (GGGG/GT). The use of the distal out-of-frame splice site caused a reading-frame shift of E5 downstream, which, in turn, overread the original termination codon and utilized a new ("delayed") termination codon further downstream. Overall, the original splice variant and GH-V/Δ4 shared 124/219 and differed by 95/219 amino acids.

**Figure 3:**
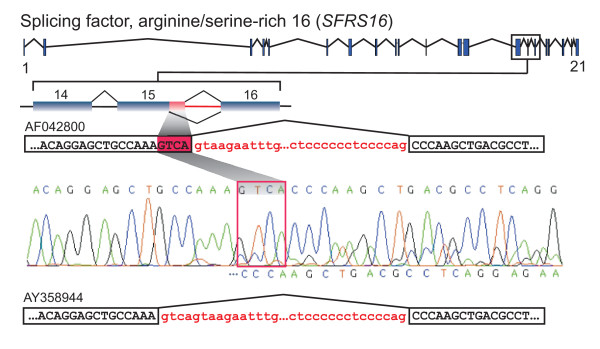
**Experimental validation of a tandem donor activated in E15 of the *SFRS16 *gene using RT-PCR and direct sequencing.** The top shows the gene structure of *SFRS16*; in the middle and bottom, E14-16 are schematically extracted and the 3'-end core and full extension sequence of E15 for proximal (TCA/gtaaga) and distal (AAA/**gtca**gt) splicing are shown. Prior to reaching the 5'ss of E15, both mRNA isoforms cannot be distinguished and consequently the electropherogram displays, for each position, one nucleotide signal peak above the base line. After the tandem donor site, two nucleotide signals above the base line become visible, indicating the presence of two isoforms.

Clearly, the detection of alternative tandem splice site exons is hampered due to the high similarity of isoforms and often only detectable by direct sequencing and protein sequence analysis. Consequently, an experimental assay was used to explore the splicing patterns of computationally identified alternative tandem donors directly. Table 1 list the names of a set of 14 genes with tandem acceptors (~8% of total), which were manually selected from known genes exhibiting a varying degree of transcript coverage (ranging from one to 35 transcripts for tandem splice site usage) and tested in a battery of human organ systems and cell types by RT-PCR primers targeted to the flanking exons; panels of nine normal tissue samples (from the brain, colon, heart, kidney, small intestine, spleen, thymus, ovary, and leukocytes) were assayed. The products of these 45 RT-PCRs were used to verify the identity of these PCR products by sequencing (see Figure 2, as well as Methods). For instance, Figure 3 shows for E15 of *SFRS16 *schematically the gene structure, proximal and distal sites of the tandem donor, and the sequence electropherogram interrogated in samples derived from the human spleen and blood. Upstream of the E15 tandem donor, both transcript sequences identically overlap and thus cannot be distinguished in the electropherogram; downstream, two nucleotide signals appear above the base line, indicating the presence of two isoforms.

**Table 1:** Summary of the experimental assay for validating computationally inferred human tandem donors.

**Ensemble gene (**ENSG00000#**)**	**Gene name**	**Region**	**PTC**	**Transcript coverage (distal/proximal)**	**Analyzed tissues**	**Confirmed donors (distal/proximal)**
172613	*RAD9A*; RAD9 homolog	CDS	+	3/17	Kidney; Leukocytes	(+/**+**); (+/**+**)
175605	*ZNF32*, zinc finger protein 32	CDS	+	14/2	Heart; Leukocytes	(**+**/+); (**+**/+)
104859	*SFRS16*; arginine/serine-rich splicing factor 16	CDS	+	9/7	Leukocytes; Spleen	(+/**+**); (+/**+**)
161574	*CCL15*, small inducible cytokine A15 precursor	CDS	+	35/6	Colon	(**+**/+)
177646	*ACAD-9*, Acyl-CoA Dehydrogenase Family, mitochondrial Precursor	CDS	+	13/8	Brain; Heart	(**+**/+); (+/**-**)
148459	*PDSS1*, Trans-Prenyltransferase	CDS	+	6/2	Small intestine	(**+**/+)
180198	*RCC1*, regulator of chromosome condensation	5'UTR	+	4/2	Small intestine; Testis	(**+**/+); (**-**/+)
170581	*STAT2*, signal transducer and activator of transcription 2	CDS	+	8/1	Brain; Thymus	(+/**-**); (+/**-**)
102878	*HSF4*, heat shock transcription factor 4	CDS	+	6/1	Colon^a^, Brain^a^	(**-**/+); (**-**/+)
090061	*CCNK*, cyclin K	CDS	+	17/1	Leukocytes	(+/**-**)
137502	*RAB30*, Ras-related Protein RAB-30	CDS	+	1/7	Leukocytes	(**-**/+); (**-**/+)
134987	*WDR36*, WD-Repeat Prtoeine 36	CDS	+	1/4	Leukocytes	(**-**/+)
157911	*PEX10*, peroxisome assembly protein 10	CDS	+	3/18	Brain	(**-**/+)
049656	*CLPTM1L*, cisplatin resistance related protein CRR9p	CDS	+	2/32	Ovary; Small Intestine	(**-**/+); (**-**/+)

A5EΔ4 splicing exons were selected according to both transcript coverage, concordance of tissues inferred from cDNA-libraries of A5EΔ4 genes, and commercially available samples. RT-PCR primers were targeted to flanking exons, assayed, and sequenced. In the last column, "+" indicates that the tested A5EΔ4 splicing exon was detected to be present in both splice variants of the corresponding samples, separately for each tested tissue (a bolded "+" indicates the major form). In all, 7/14 A5EΔ4 splicing exons were verified in panels of nine normal tissues. In the fourth column (PTC), "+" indicates the presence of a premature termination codon.^ a ^Additional retention-type intron [see Additional File 1]

Table 1 lists the outcome for all 14 genes. In all, 50 % (7 of 14 total) of selected A5EΔ4 splicing exons showed PCR-products displaying *E *= 4 nucleotides for the sets of interrogated alternative exons, and the experimentally observed splice ratio between minor and major form was in agreement with the ratio suggested by EST data. Six of seven A5EΔ4 splicing exons could be mapped to protein-coding gene sequences and all six CDS affecting alternative exons created a PTC. For human tissues samples were tried to match EST-associated cDNA libraries, using a larger battery of different organ systems and cell types might validate additional A5EΔ4 splicing exons and, therefore, conducted experiments were rather delivering a lower boundary of the presence of AS events involving tandem donors.

### Two distinct levels of A5E proximal and distal splicing

**Figure 4:**
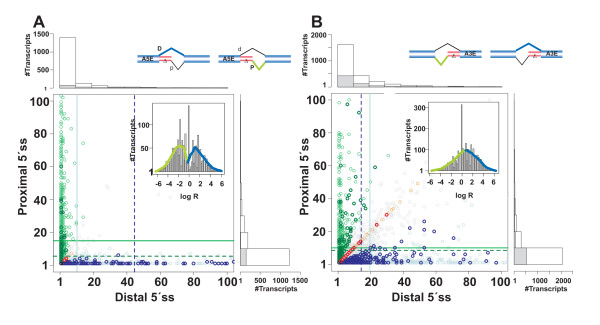
**Scatter plot of the transcript coverage of competitive and tandem donors (A) and acceptors (B). **Vertical and horizontal axes refer to the coverage of distal and proximal splice sites; solid and dotted lines mark the transcript means; A5EΔ4 and A3EΔ3 splicing exons are bolded, green and blue mark the ΔP and ΔD (major) splicing exons, respectively. The inset shows the histogram of the log-ratio (*R*) of the coverage of the distal over the proximal 5'ss (3'ss); curves marked in black show the smoothed distribution (splines, R package). In A) the coverage scatters mainly along the vertical or horizontal axis, which is indicative of preferentially including or excluding the exon extension from the core sequence. The coverage pattern was used to partition all A5Es into two main types, I and II, and a remaining type. The inset shows for the histogram of *R *a bimodal shape, which is indicative of two subpopulations of A5Es with predominant proximal or distal splice site usage. In B) the overlap between distal and proximal tandem acceptor coverage is comparatively broader, and consequently the histogram of *R *exhibits a unimodal shape consistent with a single population of A3Es.

Studies of the inclusion and exclusion of skipped exons of the human and mouse genomes have shown that SEs can be broadly subdivided into two types: SEs that are included in the majority of transcripts (termed 'major-form'), and those that are predominantly excluded ('minor-form'). Interestingly, such SEs posses different splicing and phylogenetic properties [32]. Here, we examined whether this property is more generally related to alternative exons, by analyzing the transcript coverage of 1,816 A5Es with one proximal/one distal 5'ss (no other inferred types of AS). Figure 4A shows a scatter plot of the distal against proximal 5'ss transcript coverage for both tandem and competitive donors; the individual transcript coverage of the distal (proximal) splice site is placed above (on the right-hand side). The scatter plot shows that the number of aligned transcripts ranges from a single transcripts up to more than one hundred, with the average centering on ~13, and is biased toward lower coverage (median value of 2). We defined the ratio of proximal over distal 5'ss usage (*R*) and computed *R *for human, as well as mouse, A5Es. The inset of Figure 4A shows that the histogram of the log(*R*) displays a bimodal distribution, which is indicative of the presence of two types (or subpopulations) of alternative 5'ss exons – one, which is characterized by the utilization of the proximal over the distal 5'ss (type-I), and another by the utilization of the distal over the proximal 5'ss (type-II). This is reminiscent of the "major/minor form" definition of SEs, albeit here it applies to both A5E proximal and distal splice sites. We used the threshold of *R*_c _= 2 to group all A5Es into type-I and II, or a remaining type, based on the behavior of *R *(see also Methods). Having two subpopulations of tandem donors, we denote by "PΔ4" ("pΔ4") the major (minor) form proximal donor of type-I, and by "DΔ4" ("dΔ4") the major (minor) form distal donor of type-II. Similarly, competitive proximal and distal 5'ss splice sites are denoted as "PΔ" ("pΔ") for type-I and as "DΔ" ("dΔ") for type-II, respectively (cf. Table 2).

**Table 2:** Summary of selected features analyzed for A5Es with competitive donors (A) and A5EΔ4 splicing exons with tandem donors (B), separated into major (PΔ4, DΔ4) and minor (dΔ4, pΔ4) splice forms.

**A)**				
**Features of A5Es**	**P**Δ**(major-form)**	**d**Δ**(minor-form)**	**D**Δ**(major-form)**	**p**Δ**(minor-form)**

**Number of occurrences**	872		598	
***in-frame (major-form)***	410 (47%)		257 (43%)	
***out-of-frame (minor-form)***	462 (53%)		341 (57%)	
**Mean extension length (nucleotides)**	82		119	
**Mean core length (nucleotides)**	189	107	126	245
**Transcript coverage**	3,603/19,709	324/924	2,186/13,126	330/556
**Average MAXENT score**	7.5	-0.5	6.8	4.6

**B)**				

**Features of A5EΔ4 exons**	**P**Δ**4 (major-form)**	**d**Δ**4 (minor-form)**	**D**Δ**4 (major-form)**	**p**Δ**4 (minor-form)**

**Number of occurrences**	44		118	
**Extension length (nucleotides)**	4		4	
**Mean core length (nucleotides)**	126	122	119	123
**Transcript coverage**	159/619	20/46	531/7,000	15/144
**Average MAXENT score**	7.5	2.8	7.9	-3.9

Figure 4B shows the scatter plot of the distal against proximal 3'ss transcript coverage. Here, the points are comparatively larger scattered than in Figure 4A and display an "arrow head" like structure. Using the same threshold as above, we find no clear distinction between splice sites for A3Es. Rather, the data are consistent with a single population of A3Es, and the inset shows the histogram of *R *as an approximately unimodal shape with values of *R *in a similar range as observed for A5Es.

In all, tandem and competitive A5Es comprise a set of 1,641 out of 1,868 (~88 %), remaining ~12% that either exceeded the threshold definition or were covered by a single transcript. The density of PΔ and DΔ splicing exons was ~59% (type-I) and ~41% (type-II), which was in some contrast to PΔ4 and DΔ4 of type-I with ~26% (44/171) and type-II with ~69% (118/171) exons, respectively (*P *< 0.0001; Fisher's exact test). Scatter plots, populations, and histograms were corroborated in a comparative analysis of the transcript coverage for A5Es in *M. musculus *(data not shown).

### Splice sites of A5Es score differently between type-I and type-II

**Figure 5:**
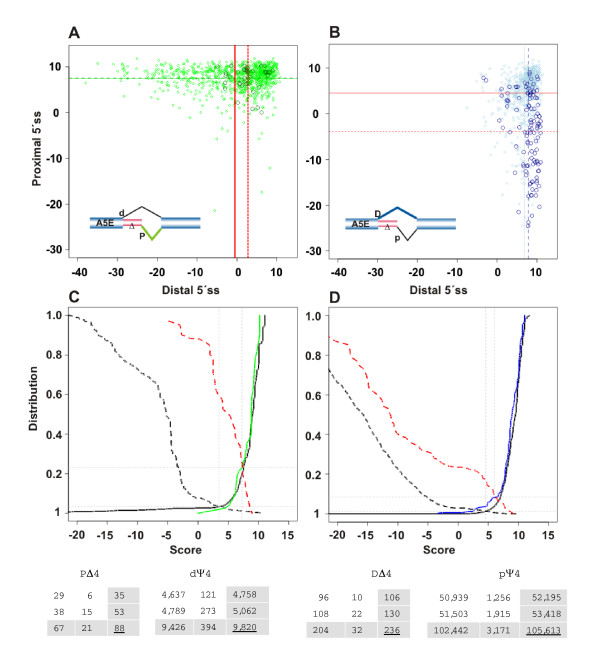
Scatter plots of 5'ss scores of competitive and tandem donors (cf. notation of Figure 4). The upper panel shows the individual and mean scores (the latter is marked by solid/dashed lines); the lower panel compares on the left-hand side the cumulative score distribution of PΔ4 and dΔ4 splice sites with constitutive 5'ss and dΨ4 (pseudo distal 5'ss, in black), and on the right-hand side pΔ4 and DΔ4 splice variants with pΨ4 and 5'ss (pseudo proximal 5'ss, in black). The threshold at which the curves intersect (S*) marks the accuracy (*A*) at which sets can be distinguished with equal classification errors on major and minor splice variants. *A*(S*) ≈ 78% for PΔ4 versus dΔ4 (PΔ4/dΔ4) and *A*(S*) ≈ 92% for pΔ4/DΔ4, and *A*(S*) ≈ 95% for dΨ4/5'ss and *A*(S*) ≈ 99% for 5'ss/pΨ4. In the bottom, tables show the number of exons of each type above and below S*; ordered table entries are: TP, FP, TN, and FN (on white background).

We computed the 5'ss score distribution to study the relationship between different types of transcript coverage and sequence-complementarity of base pairing to U1 snRNA. To this end, we applied a maximum-entropy (MAXENT), or Markov-random field, based model, which has been shown to capture additional statistical significant dependencies of splicing signals than standard position-weight matrix representations [33,34], to score the 5'ss of all A5Es (see Methods). Figure 5A shows for all PΔ and PΔ4 splicing exons of type-I the score distribution, *f*(*S*), of the distal against proximal 5'ss. The score is large (*S *> 0) when the splice site is 'close' to the consensus sequence, and small (*S *< 0) when the splice site shows marked deviations from the consensus. For type-I, we found that the scores of most PΔ and PΔ4 splicing exons were positive, ranged up to *S *= 12 (units of bit), and clustered narrowly around a mean value of *S*_PΔ _≈ *S*_PΔ4 _= 7.5 (marked by horizontal lines in Figure 5A). In contrast, scores of the corresponding dΔ and dΔ4 (the minor-forms) fluctuated more broadly, and mean values were between Δ*S*_PΔ4 _≈ 4.5 and Δ*S*_PΔ _≈ 8 weaker than the corresponding major-form splice site. Interestingly, this trend was reversed for exons of type-II (DΔ, DΔ4), where for *S*_DΔ _and *S*_DΔ4 _the score clustered between 7 to 8, yet for minor-forms was again broadly distributed and clustered around *S*_pΔ _≈ 4.6 and *S*_pΔ4 _≈ -3.9, respectively. The different pattern of narrow/broad scattering of A5EΔ4 splice site strengths in dependence of their type was corroborated in a comparative analysis of *f*(*S*) in *M. musculus *[see Additional File 1, Figure S2].

Observed patterns (/GTNN/GT) of proximal (PΔ4) and distal (DΔ4) tandem splice sites occurred with markedly different proportions (see Table 3). To what extent were the observed PΔ4 and DΔ4 splicing exons different from constitutive splicing exons (CEs) with pseudo donors having a "genomic predisposition" for tandem splicing (but were not observed)? We addressed this question by looking for constitutive 5'ss (/GT) that were flanked by another GT dinucleotide at a distance of four nucleotides either upstream (denoted as "dΨ4") or downstream of the authentic 5'ss ("pΨ4"). We searched a set of ~63,000 CEs (out of ~113,400) that exhibited proximal and/or distal pseudo tandem donors. Assuming position-independent nucleotide concentrations, the expected proportions would be ~10% (dΨ4) and ~48% (pΨ4), where the latter reflects the GT motif at positions P_5 _and P_6 _of the 5'ss consensus. We found that dΨ4 was lower than its expected occurrence and was present only in ~4% of CEs (*P *< 0.001; *z*-test), whereas pΨ4 was similar, albeit still significantly different, to the expected occurrence and present in ~47% of CEs (*P *< 0.001; *z*-test); a substantial proportion of ~5% (5,211) was comprised by GYNN/GYNNGY, but was excluded from further analysis to avoid any ambiguity. The score distribution *f*(*S*) for the above sets showed related differences. The mean scores of PΔ4 and constitutive 5'ss (downstream of dΨ4), *S*_PΔ4 _= 7.5 and *S*_5'ss _= 7.9, were about equally large (*P *< 0.13, Mann-Whitney test), yet *S*_dΨ4 _= -3.6 was significantly lower as compared with *S*_dΔ4 _= 2.8 (*P *< 2.2e-16). Similarly, the mean scores of DΔ4 and constitutive 5'ss (upstream of pΨ4), *S*_DΔ4 _= 7.9 and *S*_5'ss _= 8.7, were found to be similar, but still significantly different (*P *< 0.003), whereas *S*_pΨ4 _= -10.2 was significantly lower than *S*_pΔ4 _= -3.9 (*P *< 1.9e-13). In words, minor splice variants of tandem donors (pΔ4, dΔ4) scored larger than pseudo variants (pΨ4, dΨ4), while lower than 5'ss of constitutive splicing exons, and were consequently sufficiently different from pseudo splice sites, despite the same genomic pattern.

**Table 3:** Summary of the transcript coverage for all possible different patterns of A5EΔ4 splicing exons.

**Splice site motif**	**Distal 5'-splice site (DΔ4)**			**Proximal 5'-splice site (PΔ4)**		
		
	Occurrence	EST	cDNA	Occurrence	EST	cDNA
/GYGA/GT	36	2,555	170	3	16	8
/GYAA/GT	32	2,603	140	4	23	19
/GYAG/GT	27	922	118	19	372	75
/GYAT/GT	7	174	38	1	2	1
/GYGG/GT	6	94	18	11	91	31
/GYAC/GT	2	50	8	2	50	8
/GYCA/GT	2	5	10	-	-	-
/GYGC/GT	2	390	5	1	5	2
/GYGT/GT	2	25	13	-	-	-
/GYTA/GT	2	182	9	-	-	-
/GYTG/GT	1	-	2	3	60	15
/GYCC/GT	-	-	-	-	-	-
/GYCG/GT	-	-	-	-	-	-
/GYCT/GT	-	-	-	-	-	-
/GTTC/GT	-	-	-	-	-	-
/GTTT/GT	-	-	-	-	-	-

	**118**	**7,000**	**531**	**44**	**619**	**159**

The coverage is shown for major-form distal (DΔ4) and proximal (PΔ4) tandem donors. Genes with inferred DΔ4 splicing exons outnumber genes with PΔ4 splicing exons about 2.5-fold, which is reflected in their overall cDNA (about three-fold) and EST (about ten-fold) coverage. In addition to/GT, the/GY motif is shown, if the presence/GC was statistically significant.

### Discriminating between major and minor A5EΔ4 versus constitutive splicing exons

We used the difference between the 5'ss score distribution *f*(*S*) of major and minor A5EΔ4 splicing exons of tandem donors to test, based on the behavior of *f*(*S*) alone, how accurate PΔ4 can be distinguished from dΔ4, and DΔ4 from pΔ4 splicing exons. To this end, for type-I we computed the cumulative distribution *F*(*S*^(n)^), with *n *= 1,2,...*N*, for the set {*S*_PΔ4_}, by 1) rank-ordering all scores *S*^(n) ^from the smallest to the largest score; 2) calculating *s*_N _= Σ_m = 1..N_*S*^(m)^; and 3) normalizing *F*(*S*^(n)^) = *s*_*n*_/*s*_*N*_. By construction, *F*(*S*^(n)^) is a monotonically increasing function of *S *and takes on its largest value at *F*(*S*^(N)^) = 1. Similarly, we computed *G*(*S*) = 1 - *F*(*S*) for the set {*S*_dΔ4_}, a monotonically decreasing function of *S *that takes on its largest value at *G*(*S*^(1)^) = 1. The intersection of *F*(S*) and *G*(S*) yields for each set the accuracy at which {*S*_PΔ4_} and {*S*_dΔ4_} can be distinguished, with smallest probability of error on the classification of both sets [35,36].

Figure 5C shows for dΔ4/PΔ4 splicing exons the cumulative distributions *F*(*S*) and *G*(*S*) in the score range between -20 and 15, together with *F*(*S*) and *G*(*S*) for constitutive dΨ4/5'ss splicing exons for comparison. On the one hand, we find for PΔ4 and constitutive 5'ss that *F*(*S*) collapses to approximately one curve for *S *> 0, and that constitutive 5'ss exhibit a long range of negative scores, which was not seen for tandem donors. *G*(*S*) for dΔ4 decays similarly to dΨ4, albeit overall shifted by about ten units toward larger scores, and hence leads to a greater overlap between the *F*(*S*_PΔ4_) and *G*(*S*_dΔ4_) as compared with *F*(*S*_5'ss_) and *G*(*S*_dΨ4_) for constitutive splicing exons. Consequently, the accuracy *A*(S* = 3.5) > 95% at which one can distinguish constitutive 5'ss from dΨ4 is larger than *A*(7.3) = 78% for dΔ4/PΔ4. On the other hand, in Figure 5D we find for DΔ4/pΔ4 and constitutive 5'ss/pΨ4 similar relationships for *F*(*S*) and *G*(*S*), with *G*(*S*_pΔ4_) overall shifted by about five units toward *G*(*S*_pΨ4_). Both distributions are wider gapped than observed in Figure 5C, and thus the accuracy reached *A*(6) = 92% for alternative and *A*(4.6) = 99% for constitutive splice sites, respectively.

Note that distinguishing the sets above by means of a 5'ss score difference and the log-likelihood difference (LLD), presented in [24], are closely related. This can most easily be seen, by considering splice site scores derived from a standard position specific weight-matrix (PSWM) model with independent nucleotide frequencies: provided the PSWM background model remains unchanged, the slice site score difference is equal to the LLD. For the MAXENT splice site model incorporates higher-order statistical dependencies between nucleotides, this exact relationship is replaced by correlated values.

For this data, the subsets of pΨ4 and dΨ4 splice sites hold an upper limit on the overall number of human tandem donors, where the pseudo splice site remained unobserved or unutilized. Using the threshold scores suggested from discriminating PΔ4 against dΔ4 (S* = 7.3), as well as DΔ4 against pΔ4 (S* = 6.0), one finds that 23 (~0.5%) of the dΨ4 set and 530 (~1.0%) exons of the pΨ4 set exceed these thresholds as putatively unobserved tandem donors.

### Nucleotide conservation around major and minor A5EΔ4 splice sites

Given existing differences between tandem donors and constitutive splicing exons with either dΨ4 or pΨ4 splice sites, we compared and contrasted the nucleotide conservation around splice sites (cf. Table 4). To this end, we computed for each splice site position (P_i_) the nucleotide frequencies of proximal and distal tandem donors in type-I and type-II, and represented their information score *I *by individual sequence logos [37] (see Methods). *I *is close to zero in the absence of nucleotide conservation with respect to the background, and increases with increasing conservation up to around two bit per sequence position.

**Table 4:** Pseudo tandem donors occurring upstream (dΨ4, distal) or downstream (pΨ4, proximal) of constitutive 5'ss.

	**Constitutive exons**	**PΔ4 splicing exons **proximal, major	**A5Es **proximal, major	**DΔ4 splicing exons **distal, minor	**A5Es **distal, minor
GYNN/**GY**NNYH	4,910 (4%)	-	-	-	-
HNNN/**GY**NNGY GRNN/**GY**NNGY	52,887 (47%)	-	-	-	-
GYNN/**GY**NNGY	5,211 (5%)	-	-	-	-
HNNN/**GY**NNHY GRNN/**GY**NNHY	50,348 (44%)	-	-	-	-
/GYNN/GYNNGY	-	22 (50%)	419 (48%)	26 (22%)	235 (39%)
/GYNN/GYNNHY	-	22 (50%)	453 (52%)	92 (78%)	363 (61%)

	**113,356**	**44**	**872**	**118**	**598**

For constitutive exons, possible dΨ4 motifs are shown in rows 1 and 2, and possible pΨ4 motifs are shown in rows 3 and 4. For alternative A5EΔ4 exons, tandem donors are shown in the last two rows.

**Figure 6:**
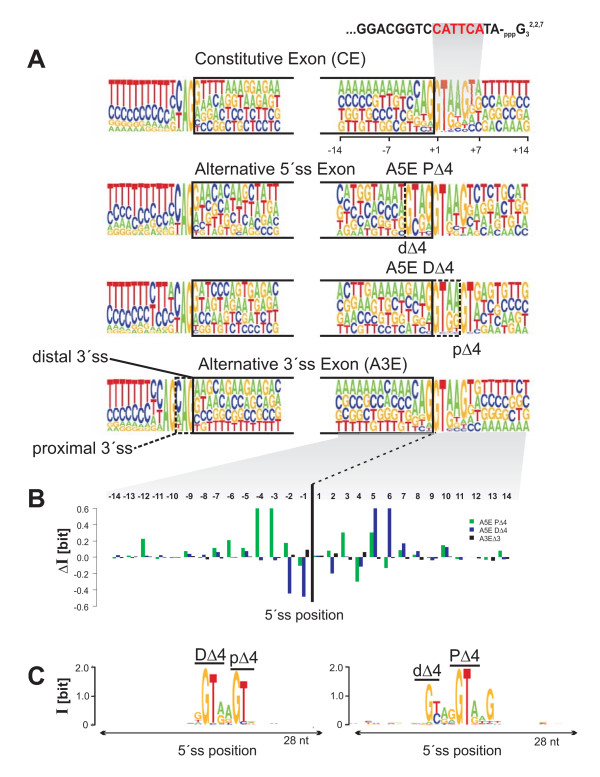
Splice site signals and sequence conservation around splice sites. A) Pictograms of 5'ss and 3'ss of constitutive, PΔ4 and DΔ4, and A3EΔ3 splicing exons. The height of a nucleotide represents the frequency of occurrence at a given position, represented in the range of 14 nucleotides around the splice junctions. Above the constitutive 5'ss, the 3'-end of the U1 snRNA is indicated. B) Information score difference (Δ*I*) between PΔ4 and DΔ4, respectively, and constitutive splicing exons, as well as A3EΔ3 and constitutive splicing exons. For each position, Δ*I *> 0 (Δ*I *< 0), indicates more (lack of) information of an alternative compared to a constitutive splice site. C) Sequence conservation of human PΔ4 and DΔ4 splice sites and splice sites of exons of orthologous mouse genes, 'anchored' at major splice sites and with > 80% exon sequence identity.

Figure 6 shows in part A) pictograms for constitutive 5'ss and 3'ss, proximal (PΔ4) and distal (DΔ4) tandem donors, as well as A3EΔ3 splicing exons; in B) the information score difference (Δ*I*) between PΔ4 and DΔ4 tandem donors to constitutive 5'ss, respectively; and in C) a species comparison of splice site positions of human A5EΔ4 splicing exons that were sequence conserved at positions P_-4_P_-3 _or P_3_P_4 _in exon of the orthologous mouse gene. We compared base frequencies of dΔ4/PΔ4 to constitutive 5'ss/pseudo dΨ4 splice sites, as well as DΔ4/pΔ4 to 5'ss/pΨ4 splice sites (data not shown), in order to identify differences in the base composition between these classes.

On the one hand, clear statistical differences were found for dΔ4/PΔ4 splicing exons with, e.g., significantly lower levels of C but higher levels of T at P_-3 _(*P *< 10^-4^, χ^2^-test) compared to dΨ4/5'ss splicing exons. Together with P_-2 _and P_-1, _which show a significant enrichment of G and A (*P *< 10^-4^, χ^2^-test) of dΔ4/PΔ4 over 5'ss/dΨ4 splicing exons, respectively, P_-2 _possibly mismatches to U1snRNA upon binding to PΔ4, while P_-3 _and P_-1 _possibly support splicing upon binding to dΔ4 due to sequence-complementarity of base pairing with U1 snRNA. Other elevated levels of dΔ4/PΔ4 splicing exons were found for T at P_-12 _(*P *< 10^-4^), A at P_-6 _(*P *< 0.05), G at both P_-5 _and P_5 _(*P *< 0.05), and C or T at P_6 _(*P *< 10^-4^, χ^2^-tests). On the other hand, DΔ4/pΔ4 splicing exons showed a significant decrease (increase) of A (T) (*P *< 0.02) worsening the match with U1 snRNA for both DΔ4 and pΔ4, while an increase of A at P_8 _(*P *< 0.01) and T at P_10 _(*P *< 0.02, χ^2^-tests) improved the U1 snRNA sequence-complementarity of pΔ4 over pΨ4. In all, several splice site positions were differently depleted or elevated, often with the possibility to enhance the sequence-complementarity to U1 snRNA [38-41]. In particular, G at position P_-1 _has been attributed as crucial for U1 but not U5 snRNA base pairing, creating stacking effects to G at P_1 _[42], and the association of P_-1 _and P_+5 _observed for A5EΔ4 major-forms, as well as A5Es and CEs but also for dΔ4 splicing exons (type-I), was pointed out in Carmel *et al*. [42]. Additionally, P_-7 _and P_-6 _of dΔ4/PΔ4 splicing exons showed elevated levels of A over dΨ4/5'ss and could promote U5 snRNA-dependent base pairing via uridines in the U5 invariant loop, suggested to compensate for weaker U1 snRNA affinity [42] (neither dΨ4/5'ss nor pΔ4 splicing exons showed elevated levels).

The different levels were in accord with the average information score that takes into account the levels of all nucleotides, at a given position, against a background level. Figure 6B shows the difference Δ*I *between tandem and constitutive 5'ss, which is positive (negative) for higher (lower) scores of tandem against constitutive 5'ss. We found that dΔ4/PΔ4 splicing exons carried overall more information at P_-12_, P_-6_-P_-2_, and P_-3_, but as well at P_-5_, whereas we found that DΔ4/pΔ4 carried less information at P_-2 _and P_-1_, but more at P_5 _and P_6_. Interestingly, Figure 6B shows no marked fluctuations of Δ*I *between tandem and constitutive 3'ss. Figure 6C supports the above positional constraints detected for type-I and type-II, by showing the conservation around major (PΔ4, DΔ4) splice sites between human A5EΔ4 splicing exons and mouse exons of orthologous genes, 'anchored' at/GT or/GC splice sites, respectively (the major site, but not the minor site, is conserved by construction). DΔ4/pΔ4 splicing exons only conserved positions P_5 _and P_6_, whereas dΔ4/PΔ4 showed two recognizable overlapping 5'ss (positions P_-4_-P_-2 _and P_1_-P_6_) and U1 snRNA sequence-complement base pairing with extension nucleotides [42].

### Exon-flanking sequences show levels of conservation in type-I, but lack of it in type-II tandem donors

Exon and flanking sequences of alternative conserved exons, or ACEs, of orthologous human and mouse genes exhibit significant levels of sequence conservation. This has most clearly been demonstrated for ACEs that undergo exon-skipping [10-12], and has also been shown for comparatively smaller sets (and thus larger statistical fluctuations) of A5Es and A3Es, including A3EΔ3 tandem acceptors [10,19]. Such conservation could imply the utilization of splicing regulatory signals that are common to orthologous sets of genes.

We examined whether A5Es and their flanking regions exhibited comparatively higher sequence conservation when compared with constitutive exons. To this end, we mapped the set of tandem and competitive A5E exons to exons of orthologous mouse genes. Imposing a level of at least 80% sequence identity and canonical splice sites, we obtained matches for about 75% of PΔ4 and 90% of DΔ4 splice variants. For each species, we extracted the sequences of exons and up to 200 nucleotides of their flanking sequences downstream of the donor splice sites, and assessed the conservation levels for exon and intron regions (cf. Table 4 and Methods). We mapped as control sets 536/653 A3EΔ3 splicing exons (1); a randomly selected subset of CEs with 4,145/4,910 and 4,082/4,910 up- (dΨ4) and downstream (pΨ4) pseudo splice sites, respectively (2); and a randomly selected subset of 2,705/4,910 SEs (3). Note that exons of orthologous mouse genes can be constitutive or alternative and, if so, of the same or a different AS type.

**Figure 7:**
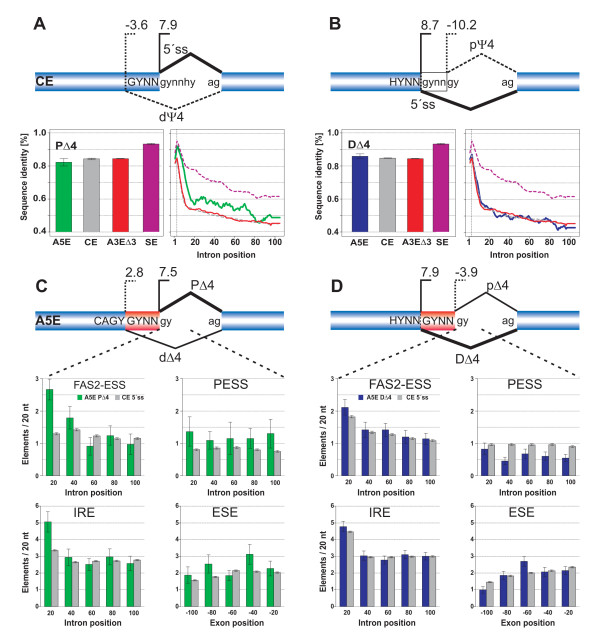
Sequence conservation and splicing regulatory elements of A5EΔ4, A3EΔ3, and SEs of orthologous human and mouse genes. Upper panels A) and B) show for different AS types graphs of the mean exon conservation and of the mean conservation of exon-flanking sequences up to 100 nucleotides downstream, respectively. The conservation is shown individually for PΔ4 (panel A, green) and DΔ4 (panel B, blue) splicing exons; extension regions of A5EΔ4 splicing exons were excluded. Lower panels C) and D) show plots of occurrences of different splicing regulatory elements, located within the first 200 nucleotides of exon-flanking sequences that share > 80% exon identity and splice site signals with mouse exons.

Figure 7A shows for PΔ4 test and control sets the exon conservation as a combined score, and the intron conservation in the range between one and 100 nucleotides. Similarly, Figure 7B shows for DΔ4 test and control sets the exon and intron conservation. Test sets have smaller overall sizes than the controls, and therefore possess larger statistical fluctuations. We observe for both exons and introns the highest level of conservation for the control set of human SEs, which exhibit a clear enrichment over tandem donor A5Es and the remaining controls, in accord with previous analyses [11,12,43]. On the one hand, we found for intron flanking regions of PΔ4 splicing exons a markedly higher level of conservation as compared with CEs, ranging up to 80 nucleotides (Figure 7A), while we found for intron flanking regions of DΔ4 splicing exons a conservation level similar to CEs (Figure 7B). On the other hand, Figure 7A and 7B show no marked differences of exon conservation levels between sequences of A5EΔ4 and the control sets (except SEs), and for all investigated exon types the average conservation level was found between 80% and 85%. Previous analyses used datasets enriched by AS events that were specifically conserved between exons of orthologous human and mouse genes (also being smaller sized [10]), and a follow-up study incorporating such data did not distinguish between PΔ4 and DΔ4 splicing exons [44].

### Occurrence of splicing signals in exon-flanking sequences

The above analyses suggested a higher downstream intron conservation of PΔ4 as compared to DΔ4 and constitutive splicing exons, in conjunction with a different splice site score between the major and minor splice variants. We examined whether the occurrence of splicing-regulatory elements could, to some extent, possibly explain the observed differences (see Methods). To this end, we searched for over-representations of known oligonucleotides (six to seven-mers) implicated in splicing regulation, which were enriched in A5EΔ4 over constitutive exon-flanking regions from one to 100 nucleotides. We made use of four sets of previously computationally and/or experimentally identified nucleic sequence elements: FAS2-ESS (A) and PESS elements (B), IREs (C), as well as ESE elements (D).

Figure 7C compares for PΔ4 splicing exons the frequency of occurrences of all four sets of sequence elements, binned to non-overlapping 20 nucleotide windows and separated for type-I and -II, against the control. Similarly, Figure 7D shows for DΔ4 splicing exons the frequency of occurrences of all four sets of sequence elements. For introns, we found for both PΔ4 and DΔ4 splicing exons a generally higher frequency of sequence elements from sets A and C, particularly from the start of the splice junction to about 40 nucleotides downstream, while elements of set B are differentially enriched in PΔ4 and suppressed in DΔ4 splicing exons. Sequence elements in exons (set D) were indicative of a general enrichment of ESEs in PΔ4 splicing exons, particularly from about 40 nucleotides upstream to the splice junction, which was not found for DΔ4 splicing exons (with a peak at about 60 nucleotides upstream the splice junction).

Exon E15 of the gene *SFRS16*, e.g., showed two purine-rich motifs, GGGGGGC and GGTGGG, located at 65 and 87 nucleotides downstream of the 5'ss (contained in sets A and B), respectively. Additional hexamers were located between the positions 117 and 123 nucleotides (GGGAGG), while other sequence elements (set C) occurred often closer to the E15 proximal donor of *SFRS16*, between five and 30 nucleotides. Poly(G)-rich sequence elements are binding sites for the family of hnRNP splicing regulators [45] and have been implicated in the control of 5'ss choice [46-48]. Interestingly, a phylogenetically conserved poly(G)-rich sequence element has previously been reported as involved in the selection of tandem/GTNNNN/GA splice sites in the splicing of the human *FGFR *gene [49].

### A5EΔ4 splicing exons often produce NMD target substrates

Inferred AS events of A5EΔ4 and A3EΔ3 splicing exons showed a "splicing dichotomy" between the 5'ss and 3'ss – while AS events of the latter result in subtle but perhaps biologically significant in-frame variation of a single amino-acid, tandem donors result in out-of-frame shifts downstream of the tandem donor and could thus lead to a truncated protein with different function or unproductive splicing, depending on the (coding) exon position. Indeed, regulated unproductive splicing and translation (RUST) has been proposed to be a mechanistic link between AS and the NMD quality control pathway [50,51]. What is the proportion of A5EΔ4 splicing exons in the present data that might be subjected to NMD? To address this, we 1) 'standardized' the initially obtained A5E annotation by matching it with REFSEQ-annotated sequences; 2) identified REFSEQ sequences with complete exon-intron structures and annotated start-stop codons of protein coding sequence (CDS) regions; and 3) imposed proximal and distal splice sites, and recalculated the altered reading-frame and stop codon position downstream of A5EΔ4 splicing exons, while neglecting possible compensating AS events at this step [see Additional File 1, Figure S3].

**Figure 8:**
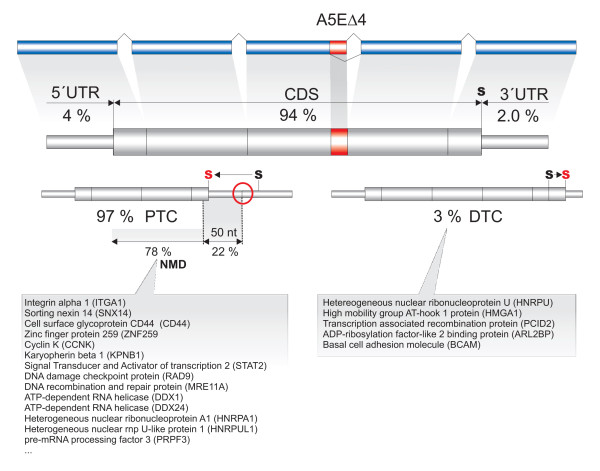
Annotation of A5EΔ4 splicing exons in REFSEQ genes. Percentages refer to fractions of A5EΔ4 splicing exons located in the 5'-UTR, coding sequence (CDS) region, or 3'-UTR. A black-colored "s" indicates the position of the stop codon relative to the REFSEQ transcript structure, whereas the red-colored version indicates the altered stop codon due to tandem donor splicing. A5EΔ4 splicing exons embedded within CDS regions are broken down into two categories, depending on the creation of a premature (PTC) or delayed termination codon (DTC). PTCs can signal mRNAs as substrates for non-sense mediated decay.

The detection of in-frame stop codons is schematically sketched in Figure 8. In all, 153/171 (~90%) inferred A5EΔ4 splicing exons were confirmed by at least one REFSEQ sequence at the distal (72%), proximal (27%) or either (1%) donor site, respectively. A large majority of A5EΔ4 splicing exons (~94%) was located in CDS regions, with only marginal proportions in the 5'-untranslated region (5'-UTR) or 3'-UTR. During splicing, choice of the out-of-frame tandem donor will create an mRNA isoform with an in-frame stop codon that introduces a premature termination codon (PTC) and shortens the C-terminus in ~97% of all considered cases. Tandem splicing of exon E8 of the human *RAD9 *gene at E8dΔ4, e.g., truncates the *RAD9 *domain by 52 amino acids (15% of total length). While possibly still maintaining the domain functionality, the loss of four C-terminal phosphoserines could prevent the interaction with the (9-1-1) cell-cycle checkpoint response complex [52]. In contexts of type-I and type-II, we found more than twice (~69 %) NMD candidates produced by DΔ4 splicing exons (where splicing of pΔ4 produced PTCs), as compared with ~26 % PΔ4 splicing exons (where splicing of dΔ4 produced PTCs). The reminder of about 5 % of NMD candidates did not stem from type-I or type-II.

In all, about three-quarters (78%) of PTCs were located more than 50 nucleotides upstream of the last exon-exon junction, and thus predicted to produce a marked proportion of NMD substrates [5]. Interestingly, a small number of A5EΔ4 splicing exons (~3%) was going to avoid the truncation of the transcript due to the out-of-frame shift but instead extended it. In close relation to premature termination codons (PTCs), we term these "delayed" termination codons (DTCs), where all detected DTCs were produced from utilization of the minor donor (pΔ4). For instance, tandem splicing at the pΔ4 donor of exon E13 of the *HNRPU *gene (ENSG00000153187), which encodes the heterogeneous nuclear ribonucleoprotein (hnRNP) U, extended the CDS region by 27 amino acids. Due to the frame shift and the occurrence of synonymous and non-synonymous codons, the amino-acid sequence is changed such that the complexity at the protein level (determined by the tool SMART [53]) increases at the C-terminal end.

## Discussion

Alternative splicing is essential for protein diversification and has recently been suggested as mechanistically linked to post-transcriptional gene regulation via nonsense mediated mRNA decay (NMD) [54]. The consequences for protein sequence and function alteration, as well as triggering of the NMD pathway, have been demonstrated for exon-skipping events in several studies [55-57]. While there is further evidence for the functioning and regulation of the remaining types of alternative exons [44], our understanding of their sequence evolution, produced AS patterns, regulation, and functioning still remains relatively vague [58]. In this paper, we analyzed differences and similarities between sets of A5Es, A3Es, and CEs, and focused on a particular type of a pair of alternative donors that are tandemly arrayed and overlapping.

Alternative 5'ss exons (A5Es) were computationally inferred from a collection of stringently aligned cDNA and EST sequences to the human genome, and their sequence features were compared to known features involved in RNA splicing. Spliced-alignments were obtained from the three independent algorithms (SIM4, BLAT, and EXALIN). EXALIN detected the smallest number of subtle AS patterns, which are characteristic of tandem donors (involving just a few nucleotides long extensions), most of which were also identified by SIM4 and BLAT. For there is no "true" method of inferring AS events, all analyses were based on the subset defined by the intersection of the predictions of all three algorithms. While one cannot rule out misalignments still arising from three methods in some instances, rigor was taken to produce a confidence-enriched set. In addition, we pursued other independent lines of evidence and experimentally validated a subset of 14 human genes with tandem donors across different tissues. The outcome confirmed about 50% A5EΔ4 splicing exons and provided evidence that a substantial fraction of tandem donors detectable in public sequence repositories are not explained by sequence alignment ambiguities. We found that almost one tenth of all human A5Es with exactly one shorter and one longer splice variant, and no other inferred splice type (SE, A3E, or RI), were A5EΔ4 splicing exons. Interestingly, Figure 1 also shows a small but persistent pattern of higher frequencies at *E *= 6, 9, 12, 15 and 18 nucleotides, which is indicative that competitive splice sites had biased extensions that preserve the reading-frame.

The central outcome of our study points to a splicing dichotomy between human alternative 5'ss and 3'ss exons in that they were markedly biased toward overlapping splice sites, with A5Es biased for *E *= 4 nucleotides (tandem donors, A5EΔ4), in contrast to A3Es biased for *E *= 3 nucleotides (tandem acceptors, A3EΔ3). Both, A3E and A5E biases in exon length variation have been previously reported [20,24,25], but their pertinent features have largely remained hidden. It is important to note that AS at both the 5'ss and 3'ss gives rise to splicing variations with very subtle changes to the encoded protein sequence, but further downstream A5EΔ4 and A3EΔ3 splicing exons lead to very different consequences. While A3EΔ3 splicing exons of the form of NAG/NAG/have been analyzed in some detail, in part with several controversial interpretations [20,24], A5EΔ4 splicing exons had not previously been confirmed experimentally and only initially been characterized [25].

In this context, pertinent questions are whether 1) such frequently observed changes arise possibly by spliceosomal error, and 2) the eukaryotic cell has found a way to neutralize or even benefit from downstream consequences that arise from such AS events. Provided their biological authenticity, what is the nature of overlapping splice site choice? Several models for splice site choice have been proposed, including the competition between antagonistic splicing factors (e.g., ASF/SF2 and hnRNP A1) and U1 snRNP [59-61], a scanning mechanism [62], or *cis*-acting motifs with different free-energy for binding U1 snRNP and splice factors between competing sites [26]. These models take into account the binding property of the U1 snRNA and additional factors. Consequently, we investigated known features involved in splice site choice, as well as consequences to the post-transcriptional regulation of A5EΔ4-carrying genes, and compared A5EΔ4 splicing exons with A3EΔ3 and constitutive splicing exons in the light of existing models for 5'ss selection.

Examined features showed differences that individually came out subtle, yet taken in concert were indicative of a spliceosomal distinction of overlapping 5'ss. We found that overlapping tandem donors, but not acceptors, can be distinguished into major-form (PΔ4, type-I; DΔ4, type-II) and minor-form (dΔ4, type-I; pΔ4, type-II) splicing exons for both proximal and distal splice sites. This is further corroborated by splice site scores, which correlated with their respective major/minor-form behavior. On the one hand, splice sites deviated most from the consensus for PΔ4 splicing exons at positions P_-4_, P_-3_, and P_3 _(Δ*I *> 0) as well as P_4_, P_5 _(Δ*I *< 0), overlapping positions of U1 snRNA nucleotides implicated in 5'ss selection [26,46]; some of which have also been related to codon preference [25]. Interestingly, more distant positions, such as P_-12 _also displayed statistically significant deviations from the consensus. Because of its close proximity to the edge of the U1 snRNA stem-loop it possibly contributes to U1 binding when dΔ4 is spliced. On the other hand, DΔ4 splicing exons showed different deviations from the consensus at P_-2, _P_-1, _P_2 _(Δ*I *< 0) as well as P_5, _P_6 _(Δ*I *< 0). Based on other experiments on position-specific stabilizing and advancing spliceosomal interactions with the 5'ss, these differences between type-I and type-II are indicative that PΔ4 improves above DΔ4 splicing compatibility with U1-snRNA,

Previous computational studies showed the conservation of sequences flanking ACEs at higher levels as compared with sequences around species-specific or constitutively spliced exons [12,63]. We observed higher levels of conservation around PΔ4, but similar levels for DΔ4 splicing exons, when compared with constitutive exons (or the 5'ss of A3EΔ3 splicing exons). Interestingly, the higher level is in accord with a larger number of detected splicing-regulatory (ESS) elements, often positioned in proximity to A5E tandem donors. In contrast to typical AS events, however, tandem donors are hindered to place regulatory elements between alternative donors. Our data show an elevation of ESE elements near dΔ4, in conjunction with an enrichment of ESS elements of flanking introns. This could be interpreted in a model, in which tandem donors restrictively exploit elements in proximal polarity (near dΔ4), to attract the U1 snRNP to this site of the tandem donor, and/or in distal polarity to dΔ4, to impair binding to PΔ4 [61].

For the majority of tandem donors was embedded in CDS regions, the downstream effects of Δ4 splicing was predictive of producing PTCs. Splicing at pΔ4 produced putative NMD substrates in more than two-thirds of all cases, whereas dΔ4 splicing exons showed about one-quarter, suggesting that pΔ4 and dΔ4 (the minor-forms) were more likely to serve as the corresponding NMD candidates. Interestingly, a small set of A5EΔ4-carrying genes avoided PTCs, yet instead was inferred to use DTCs (delayed termination codons) positioned downstream of the original signal. Utilization of the E15 proximal tandem donor of the human *SFRS16 *gene, e.g., with significantly high levels of E15 flanking sequence conservation well over 120 nucleotides in I16 (typical of RNA splicing conservation across species [12]), produced a PTC that apparently avoided NMD [64]. Using differentially binding antibodies, a previous study [30] showed that *SFRS16 *produced two detectable isoforms, which correspond to E15 tandem splicing. In another example, a Δ 4-type 5'ss change from type-I (wild-type) to type-II splicing was observed in E10 of human patients with a deficiency in the adenosin deaminase (*ADA*) gene, where a P_+1_G>A transition downstream of E10 activated splicing of a latent proximal donor [65].

A survey of gene ontology (GO) functions of the categories "molecular function" and "biological process" for genes with PΔ4 and DΔ4 splicing exons showed a significant enrichment in several proteins, while after corrections for multiple testing only the single GO-term "RNA binding" (*P *< 0.005, *t*-test) was significantly enriched, when compared between PΔ4 and dΨ4, as well as DΔ4 and pΨ4, splicing exons (see Methods).

## Conclusion

This study substantially affirms the utilization of tandem donors, thus supporting and complementing earlier findings of previously undetected AS events [25,44]. While there exist examples of cryptic Δ 4-type 5'ss in the literature [33,66], here we demonstrated that such splice variations are potentially enriched in authentic AS events, also supported by experimental studies [30,67]. Critically, pertinent data are not yet at hand to make conclusive inference about the specific regulation of A5EΔ4 splicing exons (e.g. controlled expression of species-specific minor/major isoforms), here transcript data acquisition and careful spliced-alignments have added to a higher confidence of tandem donor (and acceptor) utilization, and deeper insight will require different types of data, e.g., from mini-genes in different organ systems and cell types, U1 snRNP mutants, or variations of splicing factor dosages.

In one extreme view, incorporating a mechanistic and dosage-dependent model [26,61], the selection of AS sites depends on the properties of U1 and/or U6 snRNPs binding interrelated with antagonistic effects mediated by splicing enhancing and suppressing factors. Thus it was shown, e.g., that the choice of a tandem splice site of E10 of the *FGFR *gene can be determined by a higher sequence-compatibility of the E10 proximal splice site (pΔ6) to U6 snRNA [49]. In addition, constraints set by secondary mRNA structures [68,69] have been shown to influence splice site choice. In the opposite extreme, suggested by the reduced difference of splice site scores, tandem donors could be the outcome of stochastic binding at overlapping 5'ss and lack implicit functional implications [24], which is supported by type-I isoforms. Either view largely requires the NMD pathway to control deliberatively or aberrantly produced truncated messages.

Coming back to the question of whether there is a possible benefit of generating flawed mRNA isoforms, by deliberately or aberrantly produced AS variants with out-of-frame shifts and PTCs (either due to A5EΔ4 or other types of AS), what could be their functional utilization on the transcriptional or translational level? If such splice variants would be generally produced across organ systems and cell types, in addition to their normal splice variants, cells would have means of producing low levels of imperfect proteins. Depending on the efficiency of mRNA quality control, a fraction of which is subjected to the NMD pathway during the first pioneer round of translation and degraded, while a remaining fraction could still misfold and – depending on the quality control of protein synthesis – form defective ribosomal products (DRiPs). Ubiquitin-tagged peptide fragments that originate from DRiPs have recently been identified as a potent source of antigens for display by the MHC class I molecules on the cell surface to cognate CD8^+ ^T-cells, in agreement with a recently suggested mechanism of "immune surveillance" [70-72]. A motivating example is given by the human Tyrosinase-related protein 1 (*TYRP1*), which utilizes two different reading-frames to produce the protein gp75 (recognized by IgG) and a truncated 24 amino-acids long peptide. The latter was shown to be the source of an antigenic peptide specifically recognized by T-cells as a tumor rejection antigen [73]. It remains to be substantiated whether such antigenic peptides are linked to AS events that produce variants with out-of-frame shifts, such as produced by tandem donors.

## Methods

### Data set of alternative exons

Exons of human and mouse genes were extracted from the HOLLYWOOD database [23]. For two different transcripts aligned to a genomic locus, alternative 5'ss exons (A5Es) matched at their 3'ss, but exhibited exactly one short and one long splice form resulting from variation at the 5'ss. Alternative 3'ss exons (A3Es) matched at their 5'ss, but exhibited exactly one short and one long splice form resulting from variation at the 3'ss. Constitutive exons (CEs) were defined as exons of multi-exon genes that have as of date no transcript-supported evidence for undergoing any type of AS. In all AS events, A5Es, A3Es and CEs are "internal exons", and each exons had to obey the consensus splice sites/GT or/GC at the 5'ss and AG/at the 3'ss. U12-type introns were excluded from this analysis, because of their low fraction (less than 1% of the human introns).

### Spliced-alignments

Manual inspection of A5Es with short extensions (*E *< 6 nucleotides), previously excluded in HOLLYWOOD, revealed a substantial amount of putative alignment artifacts due to misaligned nucleotides close to exon-intron junctions [see Additional File 1]. Alignments were derived for ESTs by the SIM4 program [74], and were corroborated in a recent performance study of spliced-alignment algorithms [75]. In particular, we found examples were SIM4 introduces shifts of EST nucleotides between genomic donor and acceptor sites at genomic loci that encode short varying alternative exon (cf. Figure 1). To decrease the number of spurious alignments in the dataset of A5Es and A3Es, we used the original ESTs and created new transcript-to-genomic alignments, by utilizing two different algorithms: 1) BLAT [76], as stored in the UCSC database (see Availability and requirements section for URL); and 2) EXALIN [75], with the parameter set (m, n, q, r, x) = (25, 25, -25, -25, and -25). Manual inspection of control samples in the alignment results confirmed a clearly improved quality in the correct exon-intron boundary recognition. In all, about 35% of all initial A5E predictions (~9 %) of A5EΔ4 splicing exons could be confirmed by both BLAT and EXALIN alignments. Subsequent analyses were performed using the subset confirmed by three alignment methods.

### Classification of major and minor tandem donors

The number of transcripts that aligned either to the distal *N*(d) or proximal *N*(p) donor was used to classify A5Es. To this end, one can 1) calculate the ratio *R *(0 < R ≤ 1) of the lower over the higher transcript coverage as *R *= *N*(d)/*N*(p), if *N*(d) <*N*(p), or 1/*R *if *N*(p) <*N*(d); 2) compute the overall number of A5Es below a threshold value, *R *<*T *(0 < T < 1); and 3) define A5Es as "major" if the transcript coverage was at least twice as large as the corresponding "minor" splice site (*T *= 0.5). In this analysis, the threshold for minimal coverage was taken as a single transcript.

### Statistical analysis of splice site

The deviation of splice sites from the consensus was quantified by a maximum-entropy scoring model, implemented in MAXENTSCAN and publicly available [34]. The 5'ss model incorporates the last three (first six) nucleotides of the exon (intron), and the 3'ss model incorporates the last 20 (first three) nucleotides of the intron (exon). Sequence logos and pictograms were computed and displayed using the WEBLOGO tool with finite-sample size correction [37].

*P*-values of splice site frequencies where calculated as follows: 1) frequencies of occurrences at the considered at PΔ4 and pΨ4 splicing exons, as well as DΔ4 and dΨ4 splicing exons, where compared by a 4 × 2 contingency table and χ^2^-test; 2) statistically significant positions were selected at *P *< 0.05; 3) at the same position, the nucleotide (maximally two nucleotides) with the largest difference of the frequency of occurrence between two types (e.g., PΔ4 and pΨ4) was subsequently tested against the remaining nucleotides by 2 × 2 contingency table and χ^2^-test, where *P *< 0.05 was considered as statistically significant.

The information along a sequence was calculated as the relative (or Kullback-Leibler) entropy, which estimates the "distance" between an observed frequency distribution (*p*) to an expected frequency distribution (*q*), according to [77]

$$I=\sum_{k}p_{k}\cdot \log_{2}\left(\frac{p_{k}}{q_{k}}\right)$$

where *k *denotes the number of possible outcomes. The summation over all relevant sequence positions gives the total information score. The background distribution was taken as (*q*_1_, *q*_2_, *q*_3_, *q*_4_) = {A,G,C,T} = (0.2, 0.3, 0.3, 0.2).

### Identification of non-sense codons

For each A5EΔ4 splicing exon, the longest cDNA that mapped to the corresponding gene with annotated CDS start and end position was taken as a reference sequence. In most cases such a reference was only available for either the proximal or distal alternative splice form. Identification of mRNAs with the potential to trigger NMD was performed, by comparing the reading-frame after splicing at each tandem donor. Tandem events led to a new reading-fame, the first downstream non-sense codon of which was detected and analyzed for PTCs occurring more than 50 nucleotides upstream of the last exon-exon junction to elicit NMD [5,50].

### Detection of sequence conservation

The core of A5EΔ4 splicing exons was matched against mouse genomic DNA (version mm03), using BLAST with parameter values -a2 -gT -W10 -q-2 -r3 -e0.001. Significant matches of similarity were filtered for canonical splice sites and exon-flanking regions of 200 nucleotides were extracted from the genomic sequence. Subsequently, orthologous human and mouse intron regions were aligned using the DNA BLOCK ALIGNER [78], with parameter values -nomatchn -gap 0.02 -blockopen 0.2 -umatch 0.05 -pff, which detects block of conserved sequences located at possible different positions relative to splice junction. The sequence position of detected blocks of conservation was parsed and recorded with the script DBA-PARSER (Holste, unpublished data) and plotted in a region of 100 nucleotides, with a moving-average of ten nucleotides. Exon conservation was determined by the score (*S*_ort_) from CLUSTALW alignments, self-alignment of the larger exons to yield the score *S*_id_, and calculation of the normalized score S_tot _= S_ort_/S_id_.

### Experimental assay

1) RT-PCR amplification: For validation of splice variants, nested PCR was performed using 100 ng cDNA templates from the Human Multiple Tissue cDNA Panels I and II (BD Biosciences). Splice variants were enriched for EST originating from different cDNA libraries and, for a given gene, suitable tissues were chosen according to the origin of ESTs for the minor splice variant or the expression profile found in the Stanford SOURCE data base [79]. Primers were obtained from Metabion. Nested RT-PCR reactions were set up with ReadyToGo PCR beads (Amersham) and 10 pmol primer in 25 μl total volume, according to the manufacturer's instructions. The thermocycle protocol was 1 min 30 sec initial denaturation at 93°C, followed by 25 cycles of 40 sec denaturation at 93°C, 40 sec annealing at 55°C, 1 min extension at 72°C, and a final 4 min extension step at 72°C. In the second round of nested PCR, 2 μl first-round product was amplified for 30 cycles. Ethanol-precipitated PCR products were directly sequenced using target-specific forward and reverse primers; 2) Sanger sequencing: Reactions were set up with 200 ng template DNA, 10 pmol primer, and BigDye v3.1 (Applied Biosystems) in 10 μl final volume, according to the supplier's instructions. The thermocycle protocol was 5 min initial denaturation at 95°C, followed by 29 cycles of 30 s denaturation at 95°C, 10 s annealing at 55°C, 4 min extension at 60°C. After ethanol precipitation, automated sequence separation and detection was done on an ABI 3730XL sequencer. Electropherograms were processed by PHRED [80]. After automated assembly (Staden package, [81]), sequence variations were verified by manual inspection using GAP4 (Staden package).

### Presence of splicing-regulatory elements

Searching for splicing regulatory elements in exon-flanking regions was performed by using the following data sets (compiled in [82]): 176 predicted exonic splicing silencers identified in Wang *et al*. [83], 753 predicted intronic enhancers and/or silcencers identified in Yeo *et al*. [29], and 1,013 putative exonic splicing silencers identified in Zhang *et al*. [84]. All elements were searched for in a region of 100 nucleotides flanking proximal tandem donors, and exact matches were counted in non-overlapping sequence windows of 20 nucleotides.

### Gene ontology (GO) annotations

GO-terms for genes with A5EΔ4 splicing exons (358 GO terms), A5Es (1,414), and CEs (3,655) were obtained from the Ensembl database (see Availability and requirments section for URL), corresponding to 129 and 1,283 genes with A5EΔ4 splicing exons or A5Es, respectively, and 8,664 genes of a control set. GO annotations for A5EΔ4 splicing exons of 129 of 166 genes (representing the total set of 171 A5EΔ4 splicing exons) were mapped, and the most frequent category annotations "molecular function" and "biological process" were selected; in decreasing order: "ATP binding", "Zinc ion binding", "Regulation of transcription, DNA-dependent", "Transferase activity", "Signal transduction", "Hydrolase activity", "RNA binding", "Protein binding", "Transcription factor activity" and "DNA binding". In order to compare the GO annotations of A5EΔ4 genes against a control, 10,000 genes with at least one pseudo splice site, dΨ4 or pΨ4 splicing exons (each comprising 129 genes) were sampled and the frequency of occurrence of a certain GO term was computed. The statistical significance (*P*-value) was calculated analogous to [29], by assessing the frequency of occurrence that a certain GO-term was present in the control more frequently than in the A5EΔ4 gene set, divided by 10,000. The outcome showed the following categories as significant as the 0.005 percent level: "Signal transduction (PΔ4/dΔ4 vs 5'ss/dΨ4, 0.07; DΔ4/pΔ4 vs 5'ss/pΨ4, 0.15), "RNA binding" (0.0004; 0.003), "GTP binding" (0.02; 0.04), "Electron transport" (0.02; 0.03), "Protein biosynthesis" (0.01; 0.03), "Signal transducer activity" (0.04; 0.08). To correct for multiple testing, we applied a (conservative) Bonferroni correction [85], divided the *P*-value chosen by the number of performed tests, and GO-terms occurring with *P*_c _< 0.05/10 = 0.005 were considered as significant.

## List of abbreviations

AS, alternative splicing or alternatively spliced; 5'ss, 5' splice site; 3'ss, 3' splice site; cDNA, complementary DNA; EST, expressed sequence tag; SE, skipped exon; A5E, alternative 5'ss exon; A3E, alternative 3'ss exon; PΔ4 (pΔ4), proximal-major (proximal-minor) tandem donor; DΔ4 (dΔ4), distal-major (distal-minor) donor; pΨ4, constitutive exon with sequence match to/GT 3', but not 5', of the splice site (and with lack of evidence of AS); dΨ4, constitutive exon with sequence match to/GT 5', but not 3', of the splice site (and with lack of evidence of AS); PTC, premature termination codon; DTC, delayed termination codon.

## Authors' contributions

DH and RB conceived the study. DH, RB, SS, and KS designed the experiments. RB performed the numerical, and SS and KS the laboratory experiments. RB, SS, KS, and DH contributed reagents/materials/analysis tools and analyzed the data. DH, RB and StS wrote the paper. All authors read and approved the final manuscript.

## Availability and requirements

Original and supplementary data files are available. Additional File 1: Figures: human exon length distribution; scatter plots of 5'ss scores of competitive and tandem donors extracted from mouse *M. musculus*; occurrences of A5E and A5EΔ4 exons in REFSEQ sequences; WEBLOGO representations of A5Es and A3Es for *E *= 3, 4,...,15 nucleotides; Tables: statistics of human and mouse cDNA/EST-to-genome alignments; statistics of MAXENT score distributions of PΔ4/pΔ4 and DΔ4/dΔ4 splicing exons; transcript coverage of all possible dinucleotides (NN) defining donors in witch the motif/GTNN/GT; sequence conservation levels for exons (CLUSTALW) and their flanking regions of 14 A5EΔ4 splicing exons assayed in RT-PCR experiments; Electropherograms). Additional File 2: A5EΔ4 splicing exons with tandem donor sequences.

Pubmed: 

UCSC: 

Ensembl database: 

## Supplementary Material

Additional file 1 **Supplementary material.** The data contain supporting figures, tables, and analysed electropherograms for the validation assay. Figures: length distribution of human exons, scatter plots of 5´ss scores of competitive and tandem donors extracted from mouse M.musculus; occurrences of A5E and A5EΔ4 exons in REFSEQ sequences; and WEBLOGO representations of A5Es and A3Es. Tables: human and mouse cDNA and EST-to-genome alignments; MAXENT score distributions of proximal major and minor (PΔ4 and pΔ4), as well as distal major and minor (DΔ4 and dΔ4), splicing exons; transcript coverage of all dinucleotides with the motif /GTNN/GT; and sequence conservation levels for exons and their flanking regions. Click here for file

Additional file 2 **Supplementary material.** The data contain inferred and classified human as well as mouse alternative exons used in this study. Part 1) human A5Es, tandem splicing exons; 2) human A5Es, major proximal forms; 3) human A5Es, major distal forms; and 4) mouse A5Es, tandem splicing exons. Click here for file
