# Supplementary material for: Comparative analysis of sequence features involved in the recognition of tandem splice sites
Source: BMC Genomics. 2008 Apr 30;9:202. doi: 10.1186/1471-2164-9-202 (PMC2423196; doi:10.1186/1471-2164-9-202)
Supplement: Additional file 1 — Supplementary material. The data contain supporting figures, tables, and analysed electropherograms for the validation assay. Figures: length distribution of human exons, scatter plots of 5´ss scores of competitive and tandem donors extracted from mouse M.musculus; occurrences of A5E and A5EΔ4 exons in REFSEQ sequences; and WEBLOGO representations of A5Es and A3Es. Tables: human and mouse cDNA and EST-to-genome alignments; MAXENT score distributions of proximal major and minor (PΔ4 and pΔ4), as well as distal major and minor (DΔ4 and dΔ4), splicing exons; transcript coverage of all dinucleotides with the motif /GTNN/GT; and sequence conservation levels for exons and their flanking regions. [file 1471-2164-9-202-S1.doc]

Comparative analysis of sequence features involved in the recognition of tandem splice sites

Ralf Bortfeldt^1^, Stefanie Schindler^2^, Karol Szafranski^2^, Stefan Schuster^1^, and Dirk Holste^3,4^

^1^ Department of Bioinformatics

Friedrich-Schiller University

Ernst-Abbe-Platz 2

D-07743 Jena

Germany

^2^ Fritz-Lipmann Institute for Aging Research

Beutenbergstraße 11

D-07745 Jena

Germany

^3^ Research Institute of Molecular Pathology

Dr. Bohr-Gasse 7

and

Institute of Molecular Biotechnology of the Austrian Academy of Sciences

Dr. Bohr-Gasse 3-5

##### A-1030, Vienna

##### Austria

**Additional File 1**

^4^ Correspondence to: [bortfeldt@minet.uni-jena.de](mailto:bortfeldt@minet.uni-jena.de) (RB) or [holste@imp.ac.at](mailto:holste@imp.ac.at) (DH)

## Content:

1. Supplementary figure, captions; p. 3
2. Supplementary tables, captions; p. 4
3. Supplementary figure; pp. 5 – 8
4. Supplementary tables; pp. 9 – 12
5. Alignment Example; pp. 13
6. Electropherograms, pp. 14 - 19

##

## Supplementary figure captions

#### Figure S1. Length distribution of human exons. Panel A), box-plots of constitutive and alternative exon lengths (median and interquartile range, “whiskers” denote the 1.5-fold interquartile range). Major-proximal (PΔ4) and major-distal (DΔ4) splicing exons, constitutive 5´ss with distal pseudo (dΨ4) and proximal pseudo (pΨ4) splicing exons, and skipped exons (SEs). Panel B), quantile-quantile-plots of exon lengths (*x*-axes, quantiles of normal distribution; *y*-axes, empirical data).

#### Figure S2. Scatter plots of 5´ss scores of competitive and tandem donors extracted from mouse *M. musculus*. Panels A) and B) shows the individual and mean scores (the latter is marked by solid/dashed lines). Scattered data and mean scores are in accord with human PΔ4 and DΔ4 splicing exons.

#### Figure S3. Occurrences of A5E and A5EΔ4 exons in refseq sequences. Each sequence was subdivided into three segments of equal size (5´, center, and 3´), and mapped A5E and A5EΔ4 splicing exons were recorded in their respective segments.

#### Figure S4. weblogo representations of A5Es and A3Es with exon extensions *E* = 3, 4,…,15 nucleotides. The middle column shows for A5Es the high degeneracy of splice sites, while the last column shows for A3Es the degeneracy of the polypyrimidine-tract, which is less (more) present with increasing extension for the proximal 3´ss (for *E* > 12 nucleotides for the distal 3´ss).

## Supplementary table captions

#### Table S1. Statistics for human and mouse cDNA/EST-to-genome alignments. The data show overall inferred human and mouse A5Es and A5EΔ4 splicing exons, genes, and the number of cDNAs and ESTs aligned. Data are given as obtained after the realignment with exalin (following the initial alignment with sim4).

#### Table S2. Statistics of maxent score distributions of proximal major and minor (PΔ4 and pΔ4), as well as distal major and minor (DΔ4 and dΔ4), A5EΔ4 splicing exons. The data are shown separately in type I (A; PΔ4, dΔ4) and type II (B; DΔ4, pΔ4).

#### Table S3. Transcript coverage of all possible dinucleotides (NN) defining donors in witch the motif /GTNN/GT. A) corresponds to type-I tandem donors, and B) corresponds to type-II tandem donors. Column 2) shows the occurrences of pseudo tandem donors. The last column shows the total transcript coverage of the major-form splice site. If the co-occurrence of /GT and /GC was statistically significant (i.e., GC more frequent than 1% of all donors as observed in the total set of human constitutive donor), then a /GY motif is stated, instead of /GT.

#### Table S4. Sequence conservation levels for exons (clustalw) and their flanking regions, using a 100 nucleotides intron segment downstream of tandem donor (dna block aligner) of 14 A5EΔ4 splicing exons assayed in RT-PCR experiments. Upper half shows the group of candidates for that a proximal and distal splice form could be reproduced, whereas for the lower half only one either the proximal or the distal splice site could be sequenced.

## Supplementary figures

#### Figure S1.

**
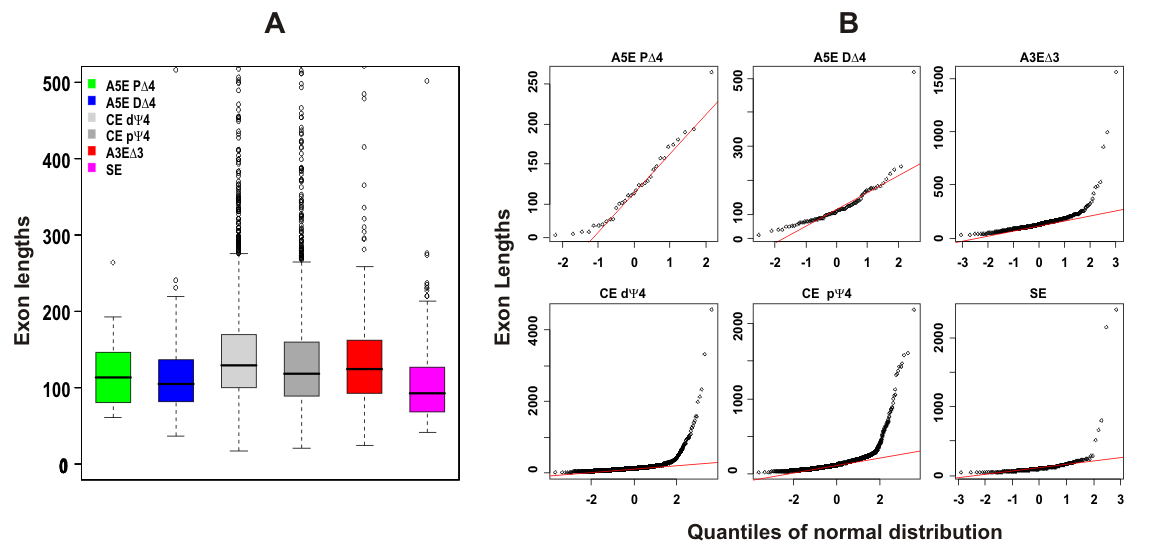
**

**Figure S2**.

####
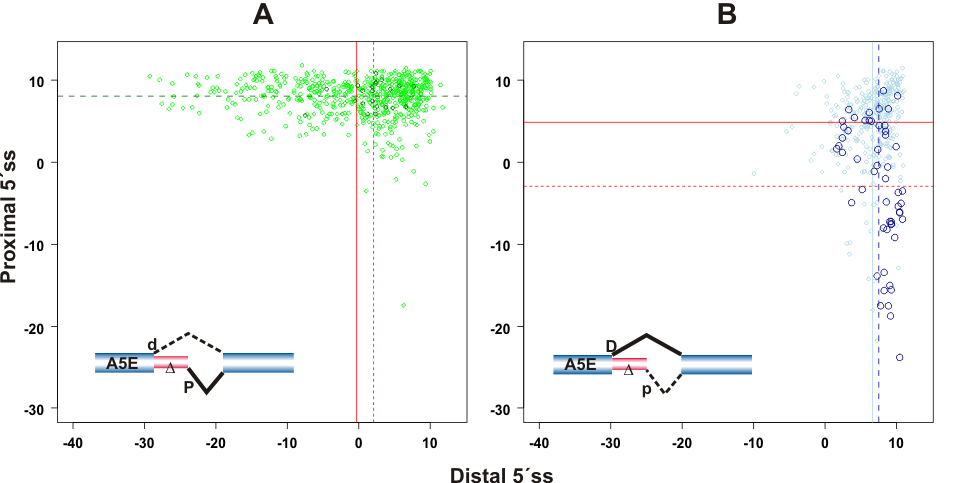


#### Figure S3.

####
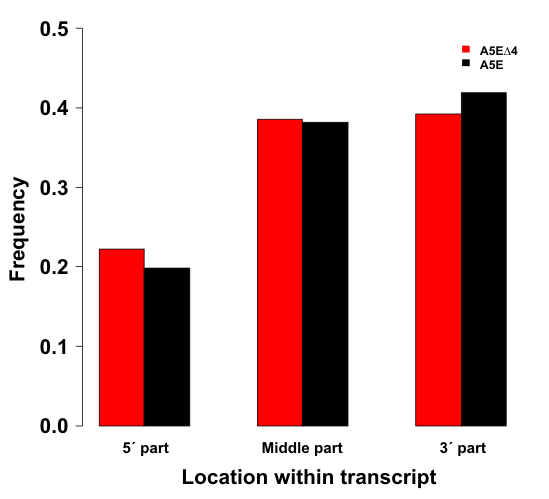


#### Figure S4.

| **Exon extension**  **[nucleotides]** | **Alternative 5**´**-splice site exon** | **Alternative 3**´**-splice site exon** |
| --- | --- | --- |
| **3** | 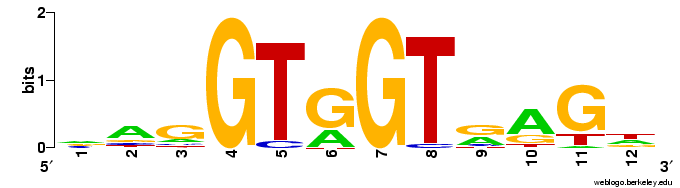 | 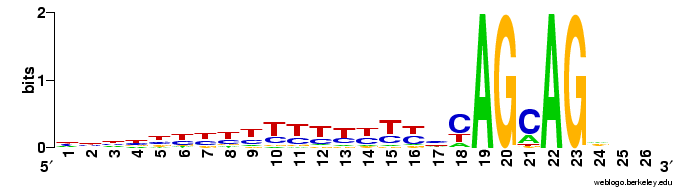 |
| **4** | 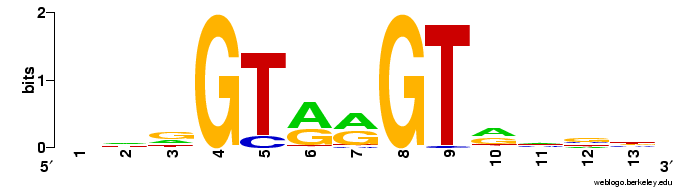 | 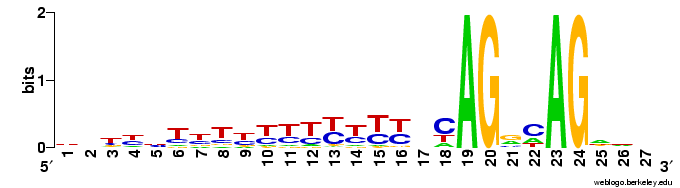 |
| **5** | 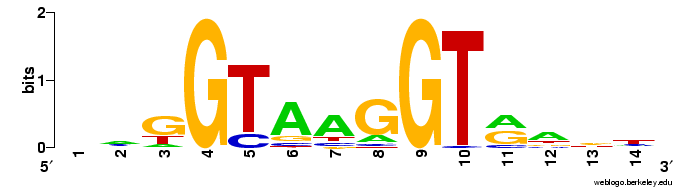 | 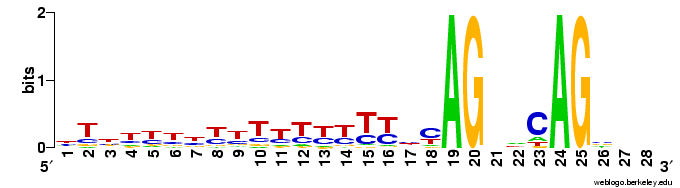 |
| **6** | 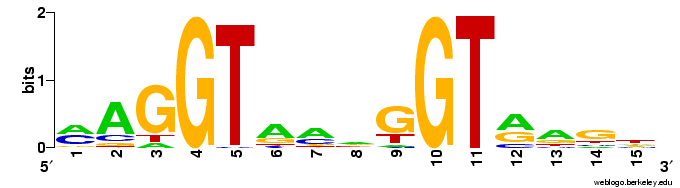 | 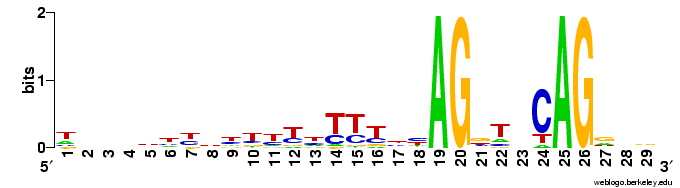 |
| **7** | 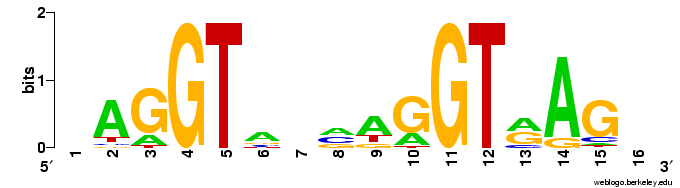 | 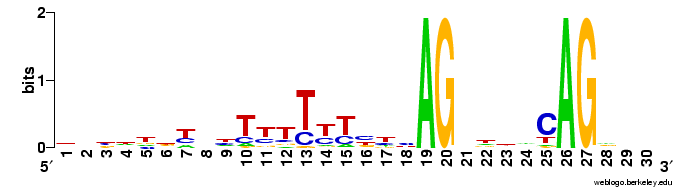 |
| **8** | 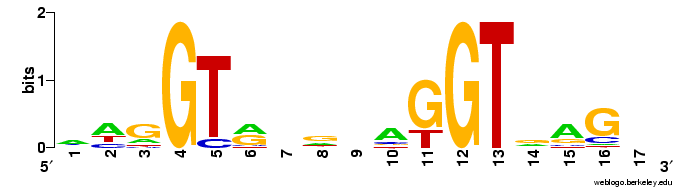 | 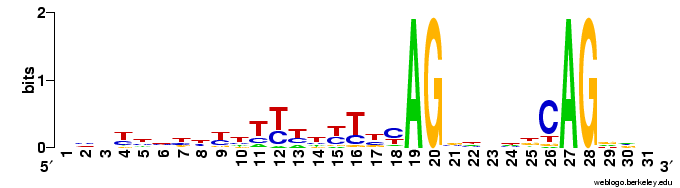 |
| **9** | 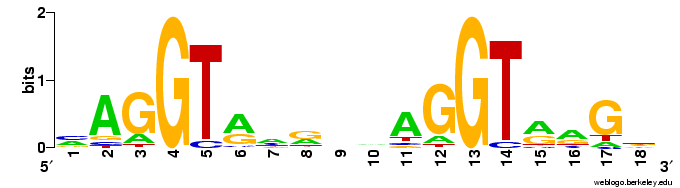 | 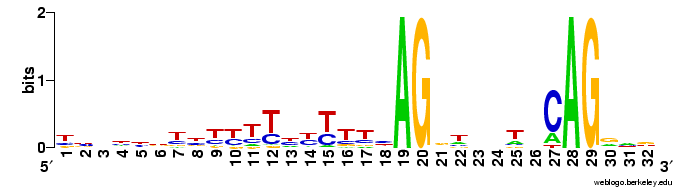 |
| **10** | 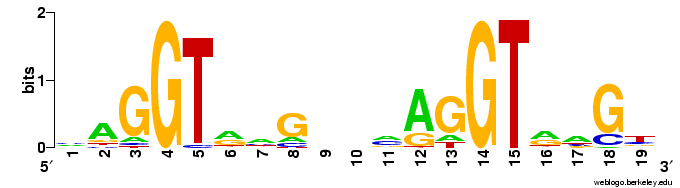 | 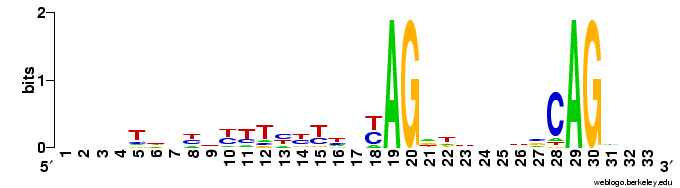 |
| **11** | 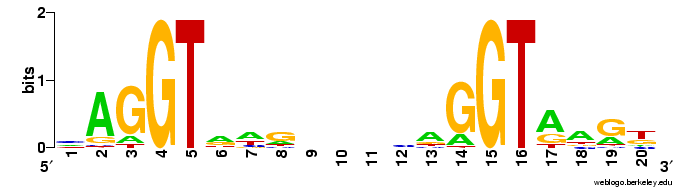 | 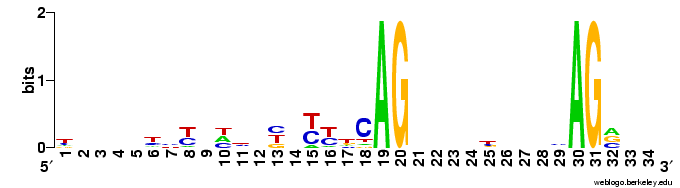 |
| **12** | 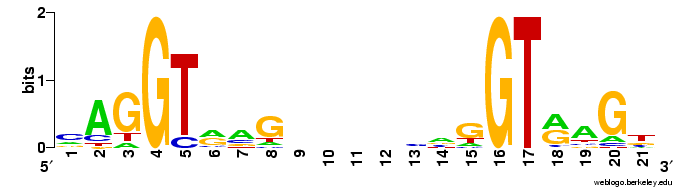 | 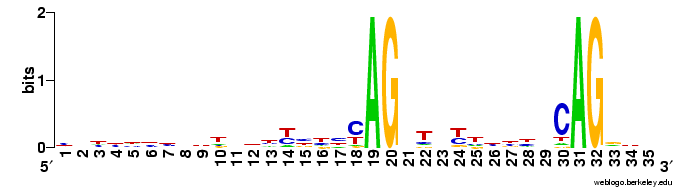 |
| **13** | 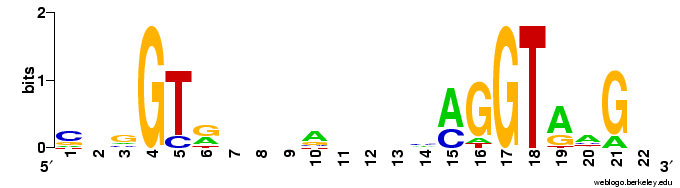 | 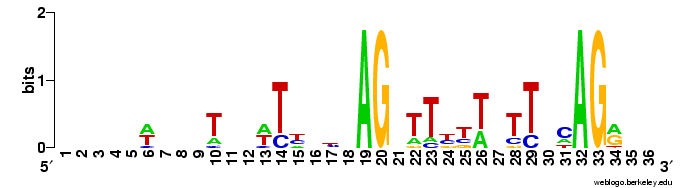 |
| **14** | 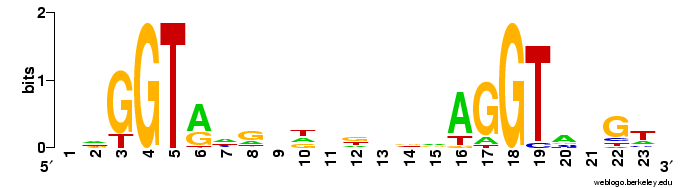 | 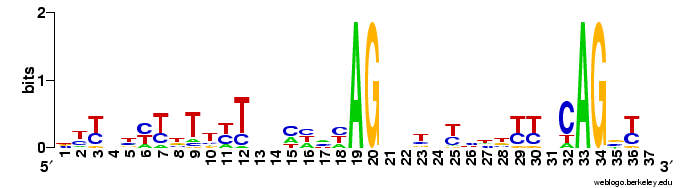 |
| **15** | 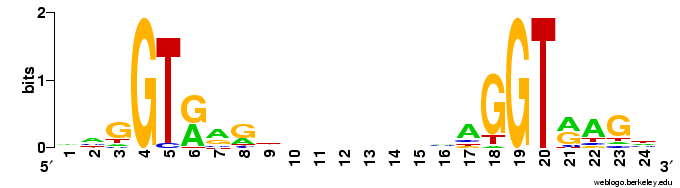 | 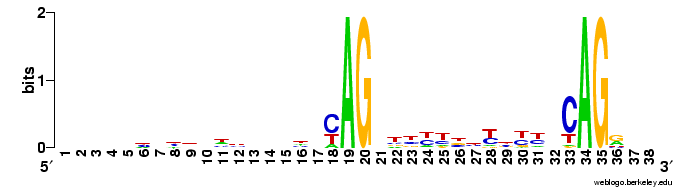 |

**Supplementary tables**

#### Table S1.

####

|  | *H. sapiens* | | ***M. musculus*** | |
| --- | --- | --- | --- | --- |
|  | **A5E** | **A5EΔ4** | **A5E** | **A5EΔ4** |
| **Exons** | 1,868 (5,275) | 171 (1,493) | 1,112 (3,185) | 81 (934) |
| **Genes** | 1,681 (4,030) | 166 (1,349) | 1,025 (2,592) | 81 (868) |
| **cDNA** | 6,874 (17,131) | 632 (5,841) | 2,016 (5,088) | 157 (1,750) |
| **EST** | 38,733 (126,480) | 6,326 (53,931) | 26,318 (83,291) | 3,969 (34,073) |

#### Table S2.

##### A

| **Class I** | **A5E/PΔ4** | **A5E/dΔ4** | **A5E/PΔ** | **A5E/pΔ** |
| --- | --- | --- | --- | --- |
| **Median** | 8.7 | -2.8 | 8.2 | 6.0 |
| **Mean** | 7.5 | -3.9 | 7.5 | 4.6 |
| **Standard deviation** | 2.6 | 8.5 | 2.9 | 5.5 |
| **Range** | (0.3 ; 10.2) | (-24.5 ; 9.1) | (-21.4 ; 11.8) | (-24.5 ; 11.8) |

##### B

| **Class II** | **A5E/DΔ4** | **A5E/pΔ4** | **A5E/DΔ** | **A5E/pΔ** |
| --- | --- | --- | --- | --- |
| **Median** | 8.6 | 2.5 | 7.6 | 2.5 |
| **Mean** | 7.9 | 2.8 | 6.8 | -0.5 |
| **Standard deviation** | 2.8 | 3.4 | 3.0 | 9.0 |
| **Range** | (-3.4 ; 11.0) | (-4.8 ; 5.4) | (-7.5 ; 11.4) | (-37.9 ; 10.8) |

#### Table S3.

##### A)

| **5´ss** | **dΨ4** | **PΔ4** | **PΔ4**  **#[cDNA]** | **dΔ4**  **#[cDNA]** | **PΔ4**  **#[EST]** | **dΔ4**  **#[EST]** | **Total PΔ4**  **coverage** |
| --- | --- | --- | --- | --- | --- | --- | --- |
| /GYAA/GT | 9/100/2/2 | 1/3/-/- | 19 | 3 | 23 | 1 | 46 |
| /GYAC/GT | 1/12/-/1 | 2/-/-/- | 8 | - | 50 | 4 | 62 |
| /GYAG/GT | 134/3509/-/16 | 6/13/-/- | 75 | 9 | 372 | 25 | 481 |
| /GYAT/GT | 1/32/1/- | 1/-/-/- | 1 | 1 | 2 | - | 4 |
| /GYCA/GT | 1/24/1/3 | - | - | - | - | - | - |
| /GYCC/GT | -/4/-/2 | - | - | - | - | - | - |
| /GYCG/GT | 43/111/-/- | - | - | - | - | - | - |
| /GYCT/GT | 10/22/-/4 | - | - | - | - | - | - |
| /GYGA/GT | 7/9/-/1 | 3/-/-/- | 8 | 3 | 16 | 1 | 28 |
| /GYGC/GT | -/1/-/- | 1/-/-/- | 2 | - | 5 | 1 | 8 |
| /GYGG/GT | 156/137/1/- | 11/-/-/- | 31 | 4 | 91 | 11 | 137 |
| /GYGT/GT | 10/3/-/- | - | - | - | - | - | - |
| /GYTA/GT | -/7/1/- | - | - | - | - | - | - |
| /GTTC/GT | -/-/-/- | - | - | - | - | - | - |
| /GYTG/GT | 125/391/-/2 | 2/1/-/- | 15 | - | 60 | 3 | 78 |
| /GTTT/GT | 5/8/1/- | - | - | - | - | - | - |
| **Total** | **4910** | **44** | **159** | **20** | **619** | **46** | **844** |

##### B)

| **5´ss** | **pΨ4** | **DΔ4** | **DΔ4**  **#[cDNA]** | **pΔ4**  **#[cDNA]** | **DΔ4**  **#[EST]** | **pΔ4**  **#[EST]** | **Total DΔ4**  **coverage** |
| --- | --- | --- | --- | --- | --- | --- | --- |
| /GTAA/GY | 15227/360/3803/42 | 29/2/1/- | 140 | 2 | 2603 | 35 | 2780 |
| /GTAC/GY | 851/14/159/4 | 2/-/-/- | 8 | - | 50 | 4 | 62 |
| /GTAG/GY | 2559/25/847/9 | 25/2/-/- | 118 | 7 | 922 | 37 | 1084 |
| /GTAT/GY | 3550/25/694/3 | 5/1/1/- | 38 | 3 | 174 | 5 | 220 |
| /GTCA/GY | 1123/11/282/5 | 2/-/-/- | 10 | - | 5 | 2 | 17 |
| /GTCC/GY | 18/1/10/1 | - | - | - | - | - | - |
| /GTCG/GY | 46/-/17/2 | - | - | - | - | - | - |
| /GTCT/GY | 199/2/54/6 | - | - | - | - | - | - |
| /GTGA/GY | 13318/18/3972/2 | 34/-/2/- | 170 | 2 | 2555 | 41 | 2768 |
| /GTGC/GY | 473/1/170/- | 2/-/-/- | 5 | - | 390 | 3 | 398 |
| /GTGG/GY | 1499/1/678/- | 5/-/-/- | 18 | 1 | 94 | 4 | 117 |
| /GTGT/GY | 631/-/124/- | 2/-/-/- | 13 | - | 25 | 4 | 42 |
| /GTTA/GY | 731/3/77/- | 2/-/-/- | 9 | - | 182 | 8 | 199 |
| /GTTC/GY | 73/-/11/- | - | - | - | - | - | - |
| /GTTG/GT | 437/1/48/3 | 1/-/-/- | 2 | - | - | 1 | 3 |
| /GTTT/GT | 591/-76/- | - | - | - | - | - | - |
| **Total** | **52887** | **118** | **531** | **15** | **7000** | **144** | **7690** |

#### Table S4.

| **Gene ID**  **(**ENSG00000#**)** | **Gene Name** | **Sequence Conservation**  **(exon / intron)** |
| --- | --- | --- |
| 172613 | *RAD9A* | 0.84 / 0.65 |
| 175605 | *ZNF32* | 0.88 / 0.61 |
| 104859 | *SFRS16* | 0.98 / 0.93 |
| 161574 | *CCL15* | 0.63 / 0.35 |
| 177646 | *ACAD9* | 0.80 / 0.33 |
| 148459 | *PDSS1* | 0.96 / 0.77 |
| 180198 | *RCC1* | - / -* |
| 170581 | *STAT2* | 0.62 / 0.72 |
| 102878 | *HSF4* | 0.83 / 0.66 |
| 090061 | *CCNK* | 0.91 / 0.45 |
| 137502 | *RAB30* | 0.92 / 0.80 |
| 134987 | *WDR36* | 0.86 / 0.71 |
| 157911 | *PEX10* | 0.78 / 0.53 |
| 049656 | *CRR9* | 0.91 / 0.53 |

- *RCC1* exon 3 (ENST00000373834) has no orthologous region within the

mouse *RCC1* locus (ENSMUSG00000028896).

## Alignment Example

A general problem of aligning cDNAs and ESTs against genomic DNA is to correctly map transcript sequences. Posttranscriptional modifications and *in vitro* transcription errors (1-4% in ESTs [Wu&Watanabe, 2005]) may affect alignment programs to output genomic coordinates of erroneously aligned transcript blocks. Subsequently, this may introduce a bias in exon datasets derived from such alignment approaches. Generally, sim4 is a very fast and capable alignment algorithm that has been successfully used for almost a decade [Florea 1998]. However, a recent comparison of alignment programs revealed apparent weaknesses in the correct alignment of mismatches or insertions/deletion near the splice junction [Zhang & Gish, 2005]. Splicing analysis is crucially depending on correct alignments of transcribed sequences, especially near alternative splice sites, and thus we applied the two best-performing programs according to a report by Zhang and Gish [2005], namely exalin and blat, for validating initial sim4 alignments. In order to demonstrate the filtering capabilities of exalin, the following examples of an erroneous and correct A5EΔ4 splice site prediction are shown for illustration below:

**A)** **Erroneous sim4 alignment**. Filtering alignments for canonical /GT and AG/ splice sites, as frequently applied to prepare data sets of alternatively spliced exons, is not sufficient to effectively remove false-positive alignments. (Incorrect aligned nucleotides are marked in red.)

| **DISTAL SPLICE SITES** | | |
| --- | --- | --- |
| **Sequence ID** | **sim4 (True positives)** | **exalin (True positives)** |
| BI037972  ENSG146592 | CAGAAGCCAAAATG AGGTTGAAGGCTGC  ||||||||||||||>>>..>>>||||||||||||||  CAGAAGCCAAAATGGTA..CAGAGGTTGAAGGCTGC | CAGAAGCCAAAATG...............AGGTTGAAGGCTGC  ||||||||||||||<<<<<<...<<<<<<||||||||||||||  CAGAAGCCAAAATGGTAAGT...TTTCAGAGGTTGAAGGCTGC |
| **PROXIMAL SPLICE SITE** | | |
| **Sequence ID** | **sim4 (False positives)** | **exalin (True negatives)** |
| BQ367677  ENSG146592 | AAATG*AGTTGGAAG* GCTGCATTGACTCA  |||||-|-|--||->>>...>>>||||||||||||||  AAATG G T AA GTA...AAGGCTGCATTGACTCA | CAGAAGCCAAAATG...............A-GTTGGAAGGCTG  ||||||||||||||<<<<<<...<<<<<<| ||| ||||||||  CAGAAGCCAAAATGGTAAGT...TTTCAGAGGTT-GAAGGCTG |

**B**) **Correct sim4 alignment**. (The Δ4 exon extension is marked in bold letters.)

| **DISTAL SPLICE SITES** | | |
| --- | --- | --- |
| **Sequence ID** | **sim4 (True positives)** | **exalin (True positives)** |
| BF871212  ENSG102878 | ACTTCATCCAGTCG GAAGAGAAGATGGA  ||||||||||||||>>>...>>>||||||||||||||  ACTTCATCCAGTCGGTA...AAGGAAGAGAAGATGGA | ACTTCATCCAGTCG...............GAAGAGAAGATGGA  ||||||||||||||<<<<<<...<<<<<<||||||||||||||  ACTTCATCCAGTCGGTAGGT...TAAAAGGAAGAGAAGATGGA |
| **PROXIMAL SPLICE SITE** | | |
| **Sequence ID** | **sim4 (True positives)** | **exalin (True positives)** |
| AW841572  ENSG102878 | CATCCAGTCG**GTAG** GAAGAGAAGATGGA  ||||||||||||||>>>...>>>||||||||||||||  CATCCAGTCGGTAGGTT...AAGGAAGAGAAGATGGA | CATCCAGTCG**GTAG**...............GAAGAGAAGATGGA  ||||||||||||||>>>>>>...>>>>>>||||||||||||||  CATCCAGTCGGTAGGTTTGT...TAAAAGGAAGAGAAGATGGA |

## Electropherograms *[[1]](#footnote-2)*


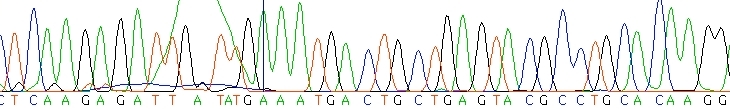


**ACAD9 brain***

GCTCAAGAGATTGATTG|AAATGACTGCTGAGTACGCCTGCACAA |GYNNGUN major

AAGAGATTGATTGGTAG|AAATGACTGCTGAGTACGCCTGCACAA GYNN|GUN minor


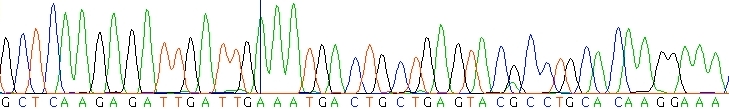


**ACAD9 heart***

GCTCAAGAGATTGATTG|AAATGACTGCTGAGTACGCCTGCACAA |GYNNGUN exclusive

AAGAGATTGATTGGTAG|AAATGACTGCTGAGTACGCCTGCACAA GYNN|GUN


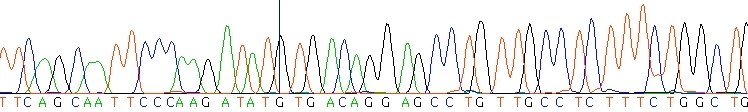


**CCNK leukocytes**

TTCAAGCAATTCCCAAGATAT|GTGA CAGGAGCCTGTTGCCTCTTT |GYNNGUN exclusive

TTCAAGCAATTCCCAAGATAT GTAA|GTGACAGGAGCCTGTTGCCT GYNN|GUN


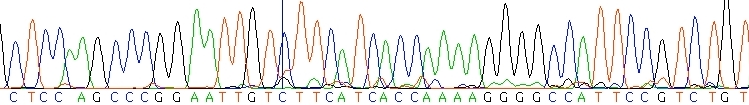


**CCL15 colon**

TGCTCCAAGCCCGGAATTGT|CTTC ATCACCAAAAGGGGCCA |GYNNGUN major

TGCTCCAAGCCCGGAATTGT GTAG|CTTCATCACCAAAAGGG GYNN|GUN minor


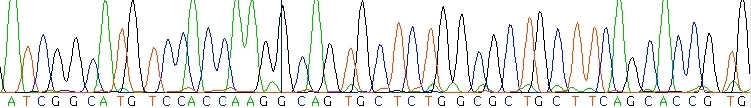


**CRR9 ovary**

GATCGGCATGTCCACCAAG|TGCT CTGGCGCTGCTTCAGCACCGT |GYNNGUN

GATCGGCATGTCCACCAAG GCAG|TGCTCTGGCGCTGCTTCAGCA GYNN|GUN exclusive


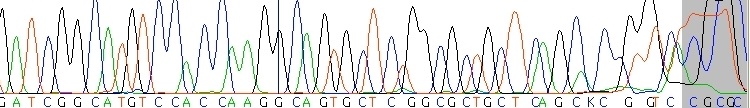


**CRR9 small intestine**

GATCGGCATGTCCACCAAG|TGCT CTGGCGCTGCTTCAGCACCGT |GYNNGUN

GATCGGCATGTCCACCAAG GCAG|TGCTCTGGCGCTGCTTCAGCA GYNN|GUN exclusive


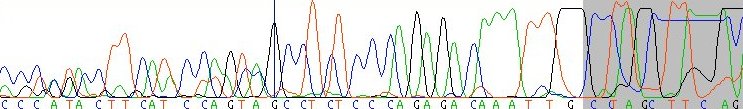


**HSF4 colon***

tgggcctagccttctacttacag|CCTCTCCCAGAGACAAAT retention 50%

constitutive exon:

AGGACCCCTACTTCATCCAGTCG|CCTCTCCCAGAGACAAAT |GYNNGUN

CCCCTACTTCATCCAGTCGGTAG|CCTCTCCCAGAGACAAAT GYNN|GUN 50%

intervening alternative exon:

CTTCTCCCTTAGACCTGGCCCAG|CCTCTCCCAGAGACAAAT |GYNNGUN

TCCCTTAGACCTGGCCCAGGTGG|CCTCTCCCAGAGACAAAT GYNN|GUN


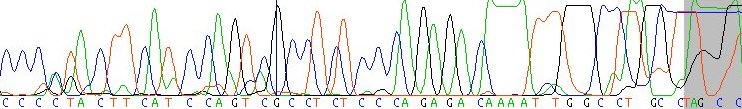


**HSF4 brain***

tgggcctagccttctacttacag|CCTCTCCCAGAGACAAAT retention minor

constitutive exon:

AGGACCCCTACTTCATCCAGTCG|CCTCTCCCAGAGACAAAT |GYNNGUN

CCCCTACTTCATCCAGTCGGTAG|CCTCTCCCAGAGACAAAT GYNN|GUN major

intervening alternative exon:

CTTCTCCCTTAGACCTGGCCCAG|CCTCTCCCAGAGACAAAT |GYNNGUN

TCCCTTAGACCTGGCCCAGGTGG|CCTCTCCCAGAGACAAAT GYNN|GUN


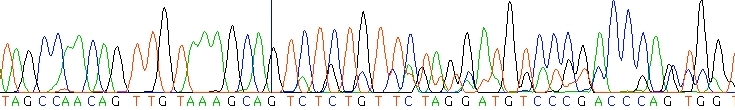


**PDSS1 small intestine**

TAGCCAACAGTTGTAAAGCA|GTCT CTGTTCTAGGATGTCCCGACCCAG |GYNNGUN major

TAGCCAACAGTTGTAAAGCA GTAT|GTCTCTGTTCTAGGATGTCCCGAC GYNN|GUN minor


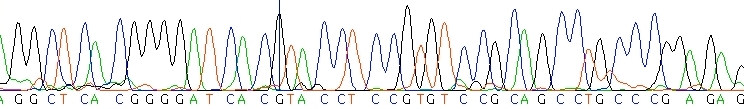


**PEX10 brain**

CAAGAGGCTCACGGGGATCAC|CTCC GTGTCCGCAGCCTGCCCGGAGA |GYNNGUN

CAAGAGGCTCACGGGGATCAC GTAC|CTCCGTGTCCGCAGCCTGCCCG GYNN|GUN exclusive


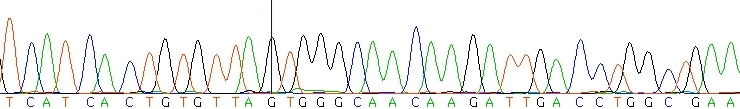


**RAB30 leukocytes**

CAACAAGGTCATCACTGTGTTA|GCAA CAAGATTGACCTGGCT |GYNNGUN

CAACAAGGTCATCACTGTGTTA GTGG|GCAACAAGATTGACCT GYNN|GUN exclusive


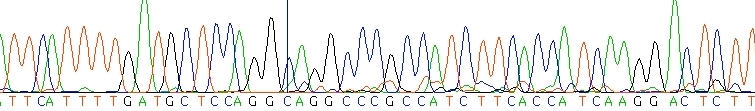


**RAD9A kidney**

GCATTCATTTTGATGCTCCAG|GCCC GCCATCTTCACCAT |GYNNGUN minor

GCATTCATTTTGATGCTCCAG GCAC|GCCCGCCATCTTCA GYNN|GUN major


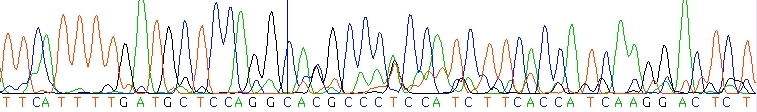


**RAD9A leukocytes**

AGCATTCATTTTGATGCTCCAG|GCCC GCCATCTTCACCATCA |GYNNGUN minor

AGCATTCATTTTGATGCTCCAG GCAC|GCCCGCCATCTTCACC GYNN|GUN major


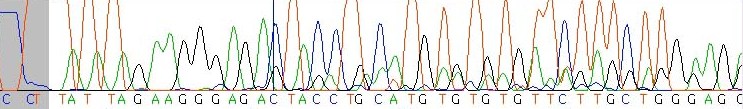


**RCC1 small intestine**

TCCTTTATATAGAAGGGAGA|CTAC ATGAATGTGTAAGATCTTGG |GYNNGUN major

TCCTTTATATAGAAGGGAGA GTAG|CTACATGAATGTGTAAGATC GYNN|GUN minor


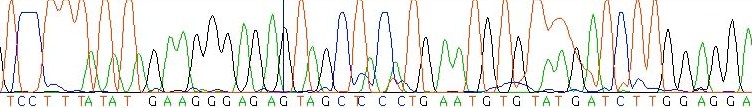


**RCC1 testis**

TCCTTTATATAGAAGGGAGA|CTAC ATGAATGTGTAAGATCTTGG |GYNNGUN

TCCTTTATATAGAAGGGAGA GTAG|CTACATGAATGTGTAAGATC GYNN|GUN exclusive


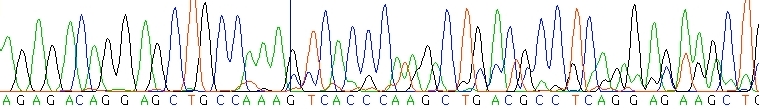


**SFRS16 leukocytes**

TAAAGAGACAGGAGCTGCCAAA|CCCA AGCTGACGCCTCAGGA |GYNNGUN minor

TAAAGAGACAGGAGCTGCCAAA GTCA|CCCAAGCTGACGCCTC GYNN|GUN major


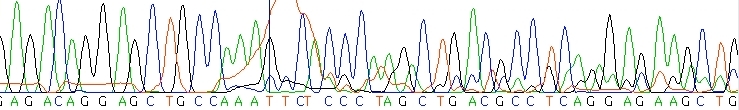


**SFRS16 spleen**

TAAAGAGACAGGAGCTGCCAAA|CCCA AGCTGACGCCTCAGGA |GYNNGUN minor

TAAAGAGACAGGAGCTGCCAAA GTCA|CCCAAGCTGACGCCTC GYNN|GUN major


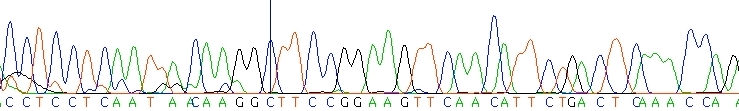


**STAT2 brain***

GAATCCTCCTCAATTACAAGG|CTTCCGGAAGTTCAACATTCT |GYNNGUN exclusive

CCTCCTCAATTACAAGGGTAG|CTTCCGGAAGTTCAACATTCT GYNN|GUN


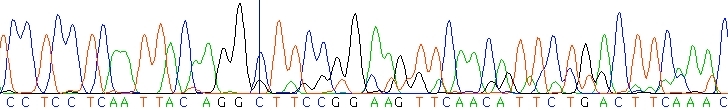


**STAT2 thymus**

GAATCCTCCTCAATTACAAGG|CTTC CGGAAGTTCAACATTCT |GYNNGUN exclusive

GAATCCTCCTCAATTACAAGG GTAG|CTTCCGGAAGTTCAACA GYNN|GUN


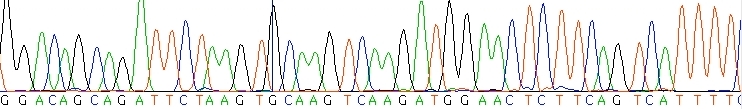


**WDR36 leukocytes**

TGGACAGCAGATTCTAAGT|GTCA AGATGGAACTCTTCAGTCATTT |GYNNGUN

TGGACAGCAGATTCTAAGT GCAA|GTCAAGATGGAACTCTTCAGTC GYNN|GUN exclusive


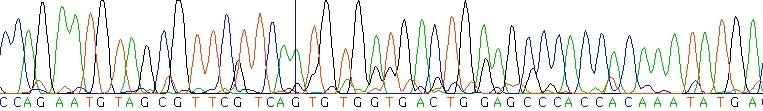


**ZNF32 heart**

CCAGAATGTAGCGTTCTTCA|ATGT GATGACTGAAGCCCACCACAA |GYNNGUN major

CCAGAATGTAGCGTTCTTCA GTGA|ATGTGATGACTGAAGCCCACC GYNN|GUN minor


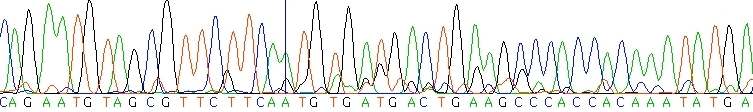


**ZNF32 leukocytes**

AATGTAGCGTTCTTCAGTGA|ATGT GATGACTGAAGCCCACCACAA |GYNNGUN major

AATGTAGCGTTCTTCAGTGA GTGA|ATGTGATGACTGAAGCCCACC GYNN|GUN minor

*****Sequencing direction downstream🡪upstream, displayed in direction of transcription

1. Sequencing direction downstream-to-upstream, displayed in direction of transcription. [↑](#footnote-ref-2)
